# Acidity-activatable dynamic hybrid nanoplatforms derived from extracellular vesicles of M1 macrophages enhance cancer immunotherapy through synergistic triple immunotherapy

**DOI:** 10.1186/s12951-024-02719-7

**Published:** 2024-07-20

**Authors:** Yawen Guo, Tingting Lv, Zijie Li, Xin Wei, Chunwang Yang, Wen Li, Xiaoming Hou, Zhiyu Wang, Ruijie Qian

**Affiliations:** 1 Department of Interventional Radiology, The First Affiliated Hospital of Zhengzhou University, Zhengzhou, Henan 450000 People’s Republic of China; 2 Department of Immuno-Oncology, The Fourth Hospital of Hebei Medical University, Shijiazhuang, Hebei 050000 People’s Republic of China; 3 Department of Ultrasound, Beijing Children’s Hospital, Capital Medical University, National Center for Children’s Health, Beijing, People’s Republic of China

**Keywords:** pH-activatable, Tumor immunotherapy, cGAS-STING pathway, Ferroptosis, Photodynamic therapy

## Abstract

Immunotherapy exhibits considerable promise for sustained tumor reduction. However, current cancer immunotherapy methods elicit limited responses due to the inadequate immunogenicity exhibited by cancer cells. This obstacle may be addressed using nanoplatforms that can activate synergistic therapies (photodynamic therapy and ferroptosis) in response to the acidic pH of the tumor microenvironment. We previously developed an amphiphilic photosensitizer, SR780, which displays satisfactory photodynamic effects. This photosensitizer is inactivated when bound to Fe^3+^ (SR780Fe) but is activated upon release in mildly acidic conditions. In this study, M1 macrophage-derived extracellular vesicles (EVs) were fused with REV and SR780Fe–loaded liposomes (REV@SR780Fe@Lip) to form REV@SR780Fe@LEV hybrid nanovesicles. Further modification with the RS17 peptide for tumor targeting enabled a combination of photodynamic therapy, ferroptosis, and cGAS-STING pathway activation, resulting in enhanced antitumor efficacy through a synergistic effect. Upon laser irradiation, REV@SR780Fe@LEV-RS17 demonstrated antitumor effects in 4T1 breast cancer models, including the inhibition of lung and liver metastasis, as well as prevention of tumor recurrence.

**Graphical Abstract:**

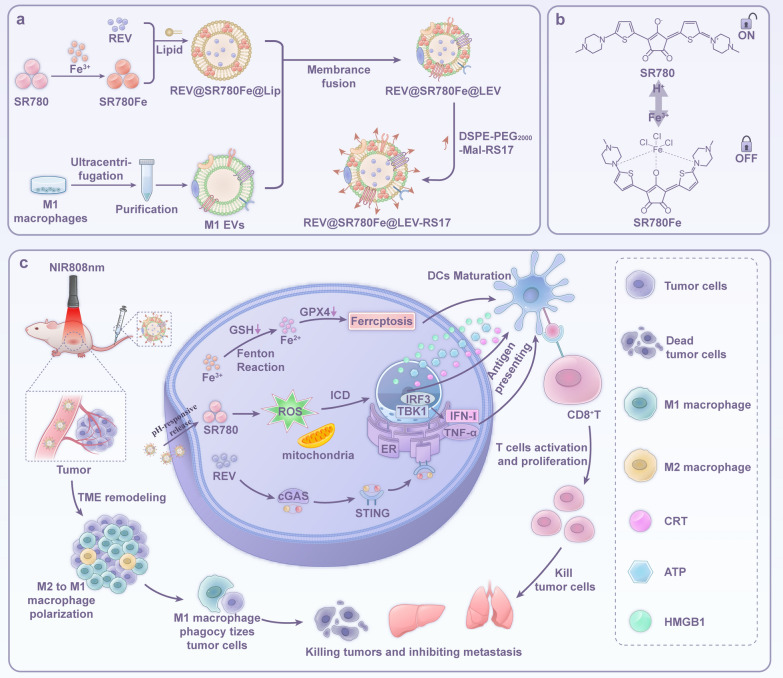

**Supplementary Information:**

The online version contains supplementary material available at 10.1186/s12951-024-02719-7.

## Background

Cancer immunotherapy, which utilizes the immune system to fight cancer, represents a new paradigm in cancer treatment [1, 2]. However, its clinical application is hindered by low patient response rates and unpredictable immune-related adverse events, such as severe neurotoxicity, cytokine release syndrome, and multiple organ dysfunction [3, 4]. By improving the pharmacokinetic and biodistribution profiles of immunotherapeutic agents and enhancing their interactions with immune cells, nanomedicines may provide safer and more efficient cancer immunotherapy [5, 6]. Nanomedicines can also simultaneously deliver multiple treatments (such as chemotherapy [7, 8], radiotherapy [9, 10], and phototherapy [10, 11]) that function synergistically with immunotherapy to provide more robust antitumor combinations [12, 13]. Activatable nanoagents are particularly effective in terms of detecting and responding to tumor microenvironment (TME) triggers (e.g., acidic pH, redox potential, enzymes, and hypoxia) [14, 15], enabling the selective release of payloads for optimal therapeutic efficacy. Thus, endogenous trigger-sensitive nanoagents have great potential for precise delivery of immunotherapeutic agents to exert potent antitumor effects.

In recent decades, numerous nanotherapeutic agents have been reported to modulate macrophage phenotypes and activate local immune responses [16]. Nanomaterials derived from natural sources, such as cell-based nanovesicles, have attracted considerable attention because of their prolonged blood circulation time, favorable biocompatibility, low immunogenicity, and appropriate size [17]. For example, M1 (i.e., M1 macrophage-derived) extracellular vesicles (EVs) inherit (from their parent cells) a natural inflammation-inducing ability that can be used to target tumors [18]. Additionally, M1 EVs demonstrate excellent potential to remodel immunosuppressive TMEs by re-educating pro-tumoral M2 macrophages towards an antitumoral M1 phenotype [19]. However, difficulties in isolation and purification, as well as inadequate cargo encapsulation, greatly affect the clinical translatability and pharmaceutical applications of M1 EVs. Conversely, synthetic nanocarriers are attractive because of properties such as controllable size, high loading efficiency, and ease of surface modification [20]. Hybrid nanovesicles, created by combining EVs with liposomes, offer a practical solution that enables EVs to exhibit tunable composition and additional features, while converting liposomes into customizable biogenic nanocarriers. In this study, we developed hybrid nanovesicles (LEV) by fusing M1 EVs with liposomes. We then modified the nanovesicles using the DSPE-PEG_2000_-Mal-RS17 peptide (RS17 peptide), a cluster of differentiation (CD)47-targeting antitumor peptide that inhibits CD47–signal regulatory protein α (SIRPα) signaling and enhances macrophage engulfment [21]. Due to the specific interactions between the RS17 peptide and CD47 on tumor cells, as well as the innate tumor-homing abilities of the M1 EVs, the manufactured hybrid nanovesicles (LEV-RS17) displayed robust tumor-targeting ability and increased drug accumulation at tumor sites.

Immune checkpoint blockade (ICB) therapy has great potential for use in the treatment of multiple cancers [22, 23]. However, its efficacy is limited to approximately 15% of patients [24] due to the “cold” properties of many tumors, which comprise low immunogenicity and minimal T cell infiltration [25, 26]., Strategies for converting “cold” tumors into “hot” tumors have been extensively investigated to enable more patients to benefit from ICB therapy. Effective techniques for this conversion include enhancing tumor cell immunogenicity by inducing immunogenic cell death (ICD) [27, 28]. During ICD, specific inducers cause endoplasmic reticulum stress, which enables the host immune system to recognize and kill cancer cells [29]. Dying tumor cells release damage-associated molecular patterns (DAMPs), including adenosine triphosphate (ATP), calreticulin (CRT), and high mobility group box 1 (HMGB1) [30–33]. The number of dendritic cells (DCs), which efficiently present tumor antigens, is positively correlated with DAMPs content [34]. Therefore, to enhance immunotherapy effectiveness, tumor cells undergoing ICD must release sufficient quantities of DAMPs. Photodynamic therapy (PDT) is a commonly used ICD inducer [35, 36]. PDT can accelerate the generation of reactive oxygen species (ROS) within mitochondria [37], resulting in oxidative damage to mitochondrial DNA (mtDNA) and triggering tumor-associated macrophages (TAMs) polarization to the M1 phenotype.

In addition to the low immunogenicity of tumor cells, evasion of the innate immune system contributes to the limited effectiveness of ICB therapy. The cyclic GMP-AMP synthase (cGAS)–stimulator of interferon genes (STING) pathway, a key component of innate immunity, has emerged as a promising target in cancer treatment [38–40]. Innate immunity is activated by the cytoplasmic cGAS enzyme upon detection of exogenous DNA in the cytoplasm [41, 42]. DNA from invading pathogens induces cGAS-mediated production of 2′-3′-cyclic-GMP-AMP (cGAMP) from ATP and GTP [41]. As a second messenger, cGAMP binds to STING [43, 44], which activates TANK-binding kinase 1 (TBK1); this pathway leads to the activation of transcription factors interferon regulatory factor 3 (IRF3) and nuclear factor κB (NF-κB). Finally, IRF3 and NF-κB elicit the production of many inflammatory cytokines, including type I interferons (IFN-I) [45]. Reversine (REV) is effective in activating cGAS-STING signaling in breast cancer [46].

During ferroptosis, iron accumulates within cells, resulting in programmed cell death [47, 48]. First, tumor cells accumulate Fe [2]^+^ through the glutathione (GSH)-mediated conversion of Fe^3+^ [49, 50]. Cell membranes are subsequently damaged by lipid peroxides formed during the Fenton reaction, which generates hydroxyl free radicals [51]. Finally, redox homeostasis is further disrupted and PDT treatment can be improved by the reduction in glutathione peroxidase 4 (GPX4) expression [52].

**Scheme 1:**
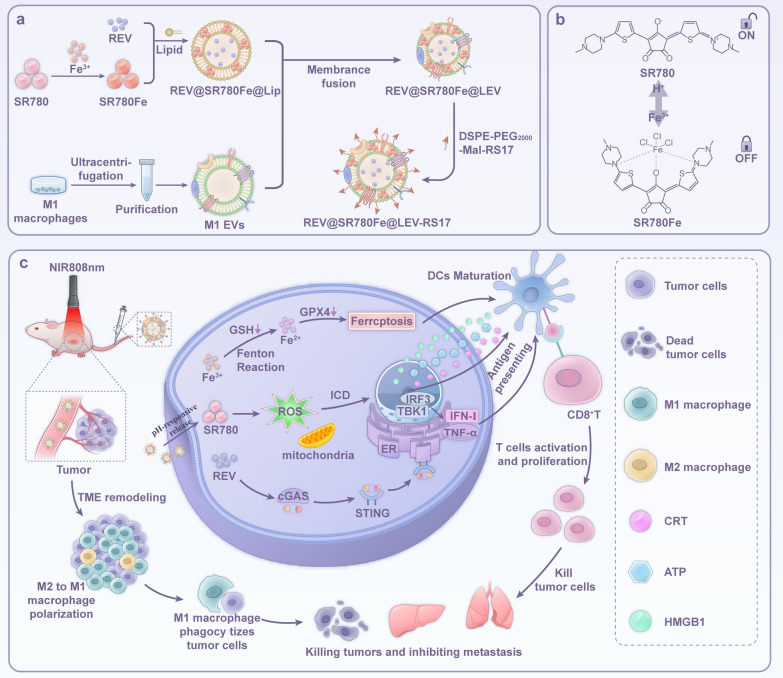
Illustration of REV@SR780Fe@LEV-RS17 NPs and their antitumor mechanisms. **a** Fabrication of REV@SR780Fe@LEV-RS17 NPs. **b** The structure conversion of SR780 and SR780Fe in Fe^3+^ and acidic environments respectively. **c** SR780Fe was activated in TME. The released SR780 produced ROS under 808 nm laser irradiation; The released Fe^3+^ ions were reduced into Fe^2+^ ions by GSH, and induce the death of cells through ferroptosis; REV activated the cGAS-STING pathway to enhance innate immune systems

In this study, we designed and synthesized an amphiphilic photosensitizer using the croconaine dye SR780, which exhibits a pH-responsive PDT effect upon excitation at 808 nm. The addition of Fe^3+^ to an SR780 solution (SR780Fe) quenches its UV absorbance, PDT effect, and fluorescence, resulting in an “off” state. Under acidic conditions, the properties of SR780 are restored, thus returning it to an “on” state. We prepared liposomes loaded with REV and SR780Fe (REV@SR780Fe@Lip). Next, we fused M1 EVs with REV@SR780Fe@Lip to form REV@SR780Fe@LEV hybrid nanovesicles. Finally, we inserted the RS17 peptide into REV@SR780Fe@LEV, forming REV@SR780Fe@LEV-RS17 nanoparticles (NPs) (Scheme 1). Due to the specific affinity of the RS17 peptide for CD47 on tumor cells and the innate homing ability of the M1 EVs, REV@SR780Fe@LEV-RS17 NPs targeted tumors and accumulated at tumor sites after intravenous administration. Then, tumor sites were irradiated using a laser with a wavelength of 808 nm. Under acidic conditions within tumor sites, degradation of the polymer SR780Fe released SR780 and Fe^3+^; this release disrupted the coordination bonds, switching SR780 from the “off” state to the “on” state. When exposed to near-infrared (NIR) photoirradiation, the activated SR780 mediated direct tumor ablation and ICD generation through photodynamic effects. Simultaneously, REV activated the cGAS-STING pathway in cancer cells and SR780Fe released Fe^3+^, which was reduced to Fe^2+^ by GSH. OH was produced in the Fenton reaction, leading to an increase in lipid peroxides and the destruction of cell membrane structure and function. GPX4 expression was indirectly inhibited through GSH consumption, disrupting the cellular redox balance and ultimately causing ferroptosis. This combined treatment, involving phototherapy, immunotherapy, and ferroptosis, activated TAMs to engulf tumor cells, promoted DCs maturation, stimulated proinflammatory cytokine secretion, and increased cytotoxic T lymphocytes (CTLs) levels at tumor sites. The resulting activation of the immune system elicited potent adaptive antitumor responses.

## Methods

### Preparation of REV@SR780Fe@LEV-RS17 NPs

REV@SR780Fe@LEV was prepared by membrane fusion technology, in accordance with previously published protocols [53]. Specifically, M1 EVs were incubated with REV@SR780Fe@Lip (1:1, v/v). To fully combine M1 EVs and REV@SR780Fe@Lip, the mixture was vortexed and sonicated for 3 min at 4 ℃. Then, the mixture was extruded 11 times using a 100-nm polycarbonate membrane filter to obtain nanosized REV@SR780Fe@LEV (Avestin, Canada). DSPE-PEG_2000_-Mal-RS17 peptide was inserted into the prepared REV@SR780Fe@LEV using post-insertion technology [54, 55]. Briefly, 1 mg of DSPE-PEG_2000_-Mal-RS17 peptide was dissolved in 1 mL of phosphate-buffered saline (PBS), then mixed with 1 mL of REV@SR780Fe@LEV solution (1 mg/mL). The mixture was vortexed, then incubated for 30 min at 4 ℃. REV@SR780Fe@LEV-RS17 NPs were obtained by centrifugation. For subsequent experiments, the REV@SR780Fe@LEV-RS17 NPs were fluorescently labeled as follows. Ten microliters of indocyanine green (ICG)–N-hydroxysuccinimide (NHS)–ester (2 mg/mL, dissolved in dimethyl sulfoxide; Xi’an Ruixi Biological Technology, China) were added to 1 mL of REV@SR780Fe@LEV-RS17 NPs and reacted under continuous oscillation for 12 h at 4 ℃ in the dark, yielding ICG-REV@SR780Fe@LEV-RS17. Unreacted ICG-NHS-ester was removed using PD-10 columns (mobile phase: PBS). The conjugation efficiency was approximately 58.4%.

### Characterization of REV@SR780Fe@LEV-RS17 NPs

Transmission electron microscopy (TEM, Thermo Scientific/Talos L120CG2, USA) was performed to determine NP size and morphology. Typical TEM parameters were accelerator: 1, column: 15, detection unit: 15, high tension: 120 kV, filament: 40, mode: high contrast, TEM bright field SA 28,000 × to 57,000 ×, beam setting: 4, focus step: 1, spot size: 3, C2 lens: ≥ 40%, and screen current: 0.5–0.8 nA. The absorption spectrum was measured using an ultraviolet–visible (UV–vis) spectrometer (PerkinElmer, USA). Dynamic light scattering (DLS) on a Malvern Zetasizer (Nano ZS, UK) was conducted to characterize the size of self-assembled NPs. Intracellular Fe^3+^ uptake was determined by atomic absorption spectrometer (PinAAcle D900, PerkinElmer, USA).

### In vitro pH-activated REV and SR780Fe release

To assess pH-activated REV and SR780Fe release, 1 mg of REV@SR780Fe@LEV-RS17 NPs was dissolved in 2 mL of either pH 7.4 or pH 5.5 release buffer and incubated at 37 ℃. At predetermined time intervals (0, 1, 2, 4, 6, 8, 12, 24 h), 100 μL aliquots of each solution were withdrawn. The released REV and SR780Fe were quantified by high-performance liquid chromatography (HPLC, UPLC SQD2 PDA, USA). Typical chromatography parameters were column: ACQUITY UPLC BEH C18 1.7 µm 2.1 × 50 mm, mobile phase: water/methanol = 2/8, flow rate: 0.45 mL/min, and absorption wavelength: 284 nm.

### Cell culture

Bone marrow-derived dendritic cells (BMDCs) were isolated from the hind limbs of female C57 mice and cultured in RPMI 1640. RAW 264.7 cells were cultured in Dulbecco’s modified Eagle medium (DMEM), whereas 4T1 cells were cultured in RPMI 1640 medium. All cells were maintained at 37 ℃ in a humidified atmosphere with 5% CO_2_. All media contained 10% heat-inactivated fetal bovine serum (FBS) and 1% penicillin–streptomycin.

### Cellular uptake

The cellular uptake of REV@SR780Fe@LEV-RS17 NPs was assessed by confocal laser scanning microscopy (CLSM, LEICA TCS SP8 STED, Germany) and flow cytometry (FCM, BD FACSymphony A5, USA). In this experiment, REV@SR780Fe@LEV-RS17 NPs were labeled with NHS-ICG. For FCM, 4T1 cells (3 × 10^5^ cells/well) were seeded in 12-well plates, incubated overnight, and then treated with ICG-REV@SR780Fe@LEV-RS17 NPs for various durations. The cells were subsequently harvested to assess the cell uptake by FCM. For CLSM, 4T1 cells (1 × 10^5^ cells/dish) were seeded in laser confocal dishes, incubated overnight, stained with 4′,6-diamidino-2-phenylindole (DAPI), and imaged by CLSM. Typical CLSM parameters were acquisition mode: xyz, format: 1024 × 1024, speed: 200 Hz, pinhole: 1 Airy unit (AU), line average: 1, gain: 800, offset: − 1, and size: 184.52 μm × 184.52 μm. Typical FCM parameters were Blue laser (488 nm): 100 mW, YG laser (561 nm): 150 mW, red laser (636 nm): 100 mW, CV: 3%, FSC: 300, SSC: 180, PE: 330, FITC: 300, APC: 350, speed: 35 μL/s, and events to record: 100,000 evt.

### Measurement of ROS production

Intracellular ROS levels were investigated using dichlorodihydrofluorescein diacetate (DCFH-DA) as a fluorescent probe. Briefly, 4T1 cells (3 × 10^5^ cells/well) were seeded in 12-well plates and cultured overnight. Next, the cells were treated for 6 h with PBS, REV@LEV-RS17, REV@SR780Fe@LEV-RS17 NPs (+, pH 7.4), REV@SR780Fe@LEV-RS17 NPs (+, pH 5.5), or REV@SR780Fe@LEV-RS17 NPs (−, pH 5.5), where (+) indicates irradiation with an 808 nm laser (0.5 W/cm^2^, 0.5 min) and (−) indicates no irradiation. The concentrations of SR780Fe and REV were both 2 μg/mL. After treatment, the culture medium was replaced with serum-free medium, and the cells were incubated for 20 min with the ROS indicator DCFH-DA (10 μM). Finally, the cells were either stained with DAPI and imaged by CLSM or harvested for FCM analysis to determine intracellular DCFH-DA levels.

### Examination of lipid peroxidation in vitro

Lipid peroxidation was assessed using BODIPY581/591-C11 (MedChemExpress, USA) and a microplate reader (SpectraMax M5e, Molecular Devices, USA). 4T1 cells (3 × 10^5^ cells/well) were seeded in 12-well plates and incubated overnight. Cells were then treated 6 h with PBS, REV@LEV-RS17, SR780Fe@LEV-RS17 NPs (+, pH 5.5), REV@SR780Fe@LEV-RS17 NPs (+, pH 5.5), or REV@SR780Fe@LEV-RS17 NPs (+, pH 7.4) with or without NIR laser irradiation (808 nm, 0.5 W/cm^2^, 0.5 min). The concentrations of SR780Fe and REV were both 2 μg/mL. After treatment, cells were stained with BODIPY581/591-C11 for 20 min and analyzed using a microplate reader, in accordance with the manufacturer’s protocol.

### Detection of mtDNA escape

Mitochondria were removed from treated cells using a mitochondria isolation kit (Beyotime, China). Nucleic acids were extracted from the remaining contents of treated cells using a nucleic acid extraction kit. Cytoplasmic 8-hydroxydeoxyguanosine (8-OH-dG) concentrations in mitochondrial and nucleic acid fractions were measured by enzyme-linked immunosorbent assays (ELISAs), in accordance with the manufacturer's protocol.

### In vitro cell viability assays

(1) Human umbilical vein endothelial cells (HUVECs), L929 cells, L-02 cells, and 4T1 cells (5 × 10^3^ cells/well) were seeded in 96-well plates and incubated overnight. Then, blank LEV-RS17 nanovesicle solutions of various concentrations (1, 5, 10, 20, and 40 µg/mL) were added to the culture medium. (2) 4T1 cells (5 × 10^3^ cells/well) were seeded in 96-well plates and incubated overnight. Next, the cells were treated with PBS, REV@LEV-RS17, REV@SR780Fe@LEV-RS17 NPs (+, pH 7.4), or REV@SR780Fe@LEV-RS17 NPs (+, pH 5.5) for 24 h. (3) Various breast cancer cell lines, including 4T1, MCF-7, and MDA-MB-231 cells (5 × 10^3^ cells/well), were seeded in 96-well plates and incubated overnight. Then, the cells were treated with REV@SR780Fe@LEV-RS17 NPs (+) and irradiated with an 808 nm laser (0.5 W/cm^2^, 0.5 min); the concentrations of SR780Fe and REV were both 2 μg/mL. Subsequently, the cells were washed and replaced with fresh medium containing 10% Cell Counting Kit-8 (CCK-8) solution; cell viability was evaluated in accordance with the CCK-8 reagent manufacturer’s protocol.

### In vitro examination of ICD

4T1 cells were treated with PBS, REV@LEV-RS17, REV@SR780Fe@LEV-RS17 NPs (+, pH 7.4), REV@SR780Fe@LEV-RS17 NPs (+, pH 5.5), or REV@SR780Fe@LEV-RS17 NPs (−, pH 5.5), where (+) indicates irradiation with an 808 nm laser (0.5 W/cm^2^, 0.5 min) and (−) indicates no irradiation. The concentrations of SR780Fe and REV were both 2 μg/mL. The culture supernatant was collected at 6 h; the levels of CRT, HMGB1, and ATP were determined using ELISA kits. For immunofluorescence staining of CRT, 4T1 cells were treated as described above, then collected and incubated with an anti-mouse CRT antibody for 2 h at 4 ℃; they were subsequently incubated with a Goat Anti-Rabbit IgG(H + L) for 1 h at room temperature. Finally, the cells were stained with DAPI and examined by CLSM. For FCM analysis of surface CRT, 4T1 cells were treated as described above, then collected and incubated with an anti-mouse CRT antibody for 30 min at 4 ℃; next, they were incubated with the second antibody for 30 min at 4 ℃. The prepared cells were analyzed using a BD A6 flow cytometer.

### In vitro activation of DCs

4T1 cells (3 × 10^5^ cells/well) were seeded in 12-well plates and incubated overnight. Next, the cells were treated for 6 h with PBS, REV@LEV-RS17, REV@SR780Fe@LEV-RS17 NPs (+, pH 7.4), or REV@SR780Fe@LEV-RS17 NPs (+, pH 5.5) and irradiated with an 808 nm laser (0.5 W/cm^2^, 0.5 min); the concentrations of SR780Fe and REV were both 2 μg/mL. The 4T1 cell supernatants were collected and added to cultures of BMDCs for 24 h. Subsequently, the BMDCs were harvested and incubated with anti-CD80 and anti-CD86 monoclonal antibodies for 30 min. The prepared cells were analyzed using a BD A6 flow cytometer. To quantify cytokine secretion in vitro, BMDCs supernatants were subjected to ELISAs measuring tumor necrosis factor (TNF)-α, IFN-β, transforming growth factor (TGF)-β, and interleukin (IL)-12 p70.

### In vitro analysis of macrophage repolarization

The macrophage repolarization efficacy of various prepared formulations was evaluated by FCM. RAW264.7 cells (M0 macrophages) were seeded in 12-well plates and stimulated with IL-4 (10 ng/mL) for 36 h to obtain an M2 phenotype. Next, the cells were incubated for 24 h with PBS, REV@LEV-RS17, REV@SR780Fe@LEV-RS17 NPs (+, pH 7.4), or REV@SR780Fe@LEV-RS17 NPs (+, pH 5.5) and irradiated with an 808 nm laser (0.5 W/cm^2^, 0.5 min); the concentrations of SR780Fe and REV were both 2 μg/mL. The cells were then collected, washed with PBS, and incubated with anti-F4/80, anti-CD206, and anti-CD86 antibodies for 30 min. Finally, cells were harvested for analysis by FCM.

### Western blot (WB) analysis

4T1 cells (1 × 10^5^ cells/well) were seeded in 6-well plates and incubated for 24 h, then divided into different groups and treated according to the experimental design. Treated cells were washed three times with PBS and lysed on ice for 30 min with radioimmunoprecipitation assay (RIPA) lysis buffer. Protein lysates were then collected by centrifugation at 12,000 rpm for 20 min at 4 ℃, and protein concentrations were determined using a bicinchoninic acid (BCA) protein assay kit. Protein samples (40 μg per lane) were separated by sodium dodecyl sulfate–polyacrylamide gel electrophoresis (SDS-PAGE) and transferred to polyvinylidene fluoride (PVDF) membranes. Membranes were blocked with 5% skim milk for 1 h, then incubated overnight at 4 ℃ with various primary antibodies (see below). Subsequently, the membranes were washed three times with Tris-buffered saline plus Tween (TBST) and incubated with secondary horseradish peroxidase-conjugated antibodies (1:5000) for 1 h at room temperature. After three additional washes in TBST, protein bands were visualized using an enhanced chemiluminescence substrate kit. An anti-glyceraldehyde-3-phosphate dehydrogenase (GADPH) antibody served as a loading control for the normalization of protein expression levels. The antibodies used in WB analysis are listed in Table S1.

### Animal model

BALB/c mice (female, 4 weeks old) were purchased from Weitong Lihua Limited Company (Beijing, China). Subcutaneous xenograft tumors were established by injecting 6 × 10^6^ 4T1 cells into the right flank of each mouse. To evaluate the ability of REV@SR780Fe@LEV-RS17 NPs to inhibit tumor metastasis, subcutaneous tumors were established on day − 10. After routine treatment on day − 1, tumors were surgically removed on day 0. For the lung metastasis model, 3 × 10^6^ 4T1 cells were injected through the tail vein; for the liver metastasis model, 5 × 10^5^ 4T1 cells were injected into the spleen. Mouse survival was subsequently monitored. All animal procedures were performed in accordance with guidelines approved by the Zhengzhou University Animal Care and Use Committee.

### In vivo biodistribution

In this experiment, REV@SR780Fe@LEV-RS17 NPs were labeled with NHS-ICG. ICG-REV@SR780Fe@LEV-RS17 NPs were intravenously administered to 4T1 tumor-bearing mice. In vivo fluorescence imaging was performed at 1, 12, and 24 h post-injection. After the final imaging time point (24 h post-injection), tumors and major organs (heart, liver, spleen, lung, and kidney) were harvested and subjected to fluorescence distribution analysis with the PerkinElmer IVIS Lumina III system.

### In vivo antitumor therapy

4T1 tumor-bearing BALB/c mice were randomly divided into five treatment groups (n = 8 each): PBS, REV@LEV-RS17, SR780Fe@LEV-RS17 NPs (+), REV@SR780Fe@LEV-RS17 NPs (−), and REV@SR780Fe@LEV-RS17 NPs (+), where (+) indicates irradiation with an 808 nm laser (1.5 W/cm^2^, 6 min) at 24 h post-injection and (−) indicates no irradiation. The concentrations of SR780Fe and REV were both 4 mg/kg. Tumor size and mouse body weight were monitored every other day for 18 days. Tumor volume was calculated as follows: volume = (length × width^2^)/2. Survival was assessed using the Kaplan–Meier method (n = 10). Synergistic therapeutic effects on lung and liver metastases were evaluated using the treatment regimen indicated in Fig. 7a. Endpoints were defined as tumor volume > 1500 mm^3^, ulceration within tumor tissue, mortality, or weight loss > 15%. Mouse blood samples were collected for cytokine analysis (i.e., TNF-α, IFN-β, TGF-β, and IL-12 p70) by ELISAs.

### Assessment of immune cell populations

To examine the immune responses induced by REV@SR780Fe@LEV-RS17 NPs, spleens and tumors were surgically removed from mice in different treatment groups. Single-cell suspensions were obtained by digestion with collagenase IV (0.3 mg/mL) at 37 ℃ for 1 h and filtration through a 70-μm mesh. Subsequently, the collected cells were blocked with CD16/CD32 antibody for 15 min, followed by staining with eBioscience™ Fixable Viability Dye eFluor™ 506 for 15 min at 4 ℃. To evaluate intratumoral CTLs content, aliquots of the collected cells were incubated with anti-CD3, anti-CD4, and anti-CD8 antibodies and analyzed by FCM, in accordance with the manufacturer’s protocol. To assess M1/M2 macrophage polarization, other aliquots of the collected cells were incubated with anti-F4/80, anti-CD206, and anti-CD86 antibodies and analyzed by FCM. Splenic CD8^+^ T cell memory was evaluated by incubating other aliquots of the collected cells with anti-CD8, anti-CD44, and anti-CD62L antibodies. Additionally, the splenic frequency of mature DCs was examined by FCM after other aliquots of the collected cells had been incubated with anti-CD80 and anti-CD86 antibodies. All samples were incubated with secondary fluorescent antibodies for detection, prior to FCM. The antibodies used in these FCM protocols are listed in Table S1.

### Immunofluorescence analysis of tumor tissue

To evaluate treatment efficacy in vivo, tumor sections were stained with hematoxylin and eosin (H&E). For immunofluorescence assays, frozen tumor tissues were sectioned at a thickness of 8 μm. Sections were incubated overnight at 4 ℃ with anti-CD3, anti-CD4, and anti-CD8 primary antibodies, then incubated with the secondary antibody and stained with DAPI. Fluorescence signals were visualized by CLSM. The primary antibodies used in this analysis are listed in Table S1.

### In vivo safety analysis of REV@SR780Fe@LEV-RS17 NPs (+)

To assess the long-term safety and biocompatibility of REV@SR780Fe@LEV-RS17 NPs, BALB/c mice (6 weeks old, 20–22 g) received a tail vein injection of REV@SR780Fe@LEV-RS17 NPs once per week for 2 months (n = 5; concentrations of REV and SR780Fe were both 10 mg/kg). At the end of the experiment, mice were euthanized and blood samples were collected for analysis of aspartate aminotransferase, alanine transaminase, alkaline phosphatase, creatinine, and blood urea nitrogen to assess renal and hepatic toxicity. Additionally, major organs (heart, liver, spleen, lung, and kidney) were harvested and subjected to H&E staining.

## Results and discussion

### Characterization of REV@SR780Fe@LEV-RS17

**Fig. 1:**
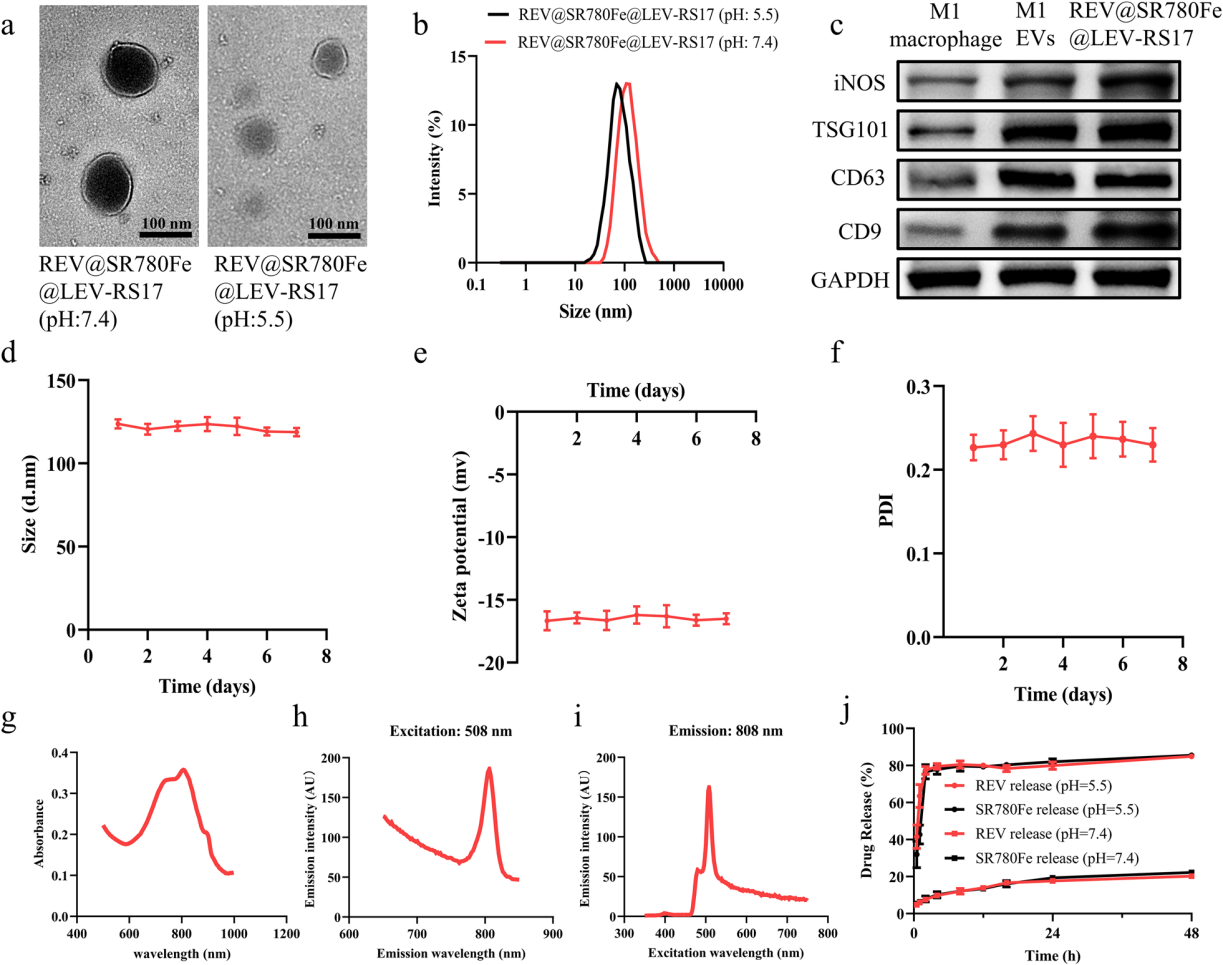
Characterization of REV@SR780Fe@LEV-RS17. TEM images (**a**) and size distribution (**b**) of REV@SR780Fe@LEV-RS17 at pH 7.4 and pH 5.5. **c** WB analysis of the protein biomarkers expression in M1 macrophage, M1 EVs and REV@SR780Fe@LEV-RS17 NPs. The variation of DLS (**d**), Zeta potential (**e**) and PDI (**f**) of REV@SR780Fe@LEV-RS17 NPs dispersed in FBS in 7 days. **g** UV–vis spectrum of SR780Fe. **h** The excitation spectra of SR780Fe emitted in 808-nm displaying the peak excitation at 508 nm. **i** The emission spectra of SR780Fe excited by 508-nm lasers displaying the peak emission at 808 nm. **j** REV release profile and SR780Fe release profile from REV@SR780Fe@LEV-RS17 in different conditions

REV@SR780Fe@LEV-RS17 NPs were prepared as shown in Scheme 1. Nuclear magnetic resonance spectrum of SR780Fe is presented in Fig. S1. The morphology and hydrated particle size of REV@SR780Fe@LEV-RS17 NPs were observed by TEM and DLS. The results showed a typical quasi-spherical shape with a lipid layer (Fig. 1a); the hydrated particle size of REV@SR780Fe@LEV-RS17 NPs at pH 7.4 was 123.8 ± 2.8 nm, with a polydispersity index (PDI) of 0.23 ± 0.02 and zeta potential of − 16.7 ± 0.7 mV, indicating an ideal size distribution (Fig. 1b). At pH 5.5, the morphology of REV@SR780Fe@LEV-RS17 NPs changed and the particle size decreased, demonstrating pH responsiveness. WB analysis of M1 EVs (Fig. 1c) revealed the presence of several exosomal marker proteins, including tumor susceptibility 101 (TSG101), CD9, and CD63 [53]. Inducible nitric oxide synthase (iNOS), a typical M1 macrophage protein marker, was also present in M1 EVs, indicating the retention of M1 EV function. After REV@SR780Fe@LEV-RS17 NPs had been incubated with FBS for 7 days, the particle size, zeta potential, and PDI values slightly fluctuated, confirming excellent stability (Fig. 1d–f). The absorption profile of SR780Fe (Fig. 1g) showed a maximum absorption wavelength of 808 nm. The excitation and emission spectra of SR780Fe are displayed in Fig. 1h and i, respectively. The release profiles of REV and SR780Fe from REV@SR780Fe@LEV-RS17 NPs under different pH conditions were investigated (Fig. 1j). At pH 5.5, approximately 77.3% ± 2.2% of REV and 76.6% ± 3.8% of SR780Fe were released within 2 h. At pH 7.4, less than 22.5% of both agents were released within 48 h. These results indicated that REV@SR780Fe@LEV-RS17 NPs were stable under physiological conditions and could achieve drug release within the acidic TME. Compared with REV@SR780Fe@LEV-RS17, the control groups REV@LEV-RS17 and REV@SR780Fe@LEV had similar particle sizes, protein markers, and stability. Due to the absence of SR780 and presence of cell membrane components alone, the morphology of REV@LEV-RS17 under TEM was comparable to that of typical exosomes (Fig. S2).

Stimuli-responsive drug delivery systems have demonstrated great potential for targeted drug delivery to tumor sites through the controlled release of therapeutic agents [56]. Several smart NPs that respond to endogenous stimuli (e.g., redox potential, enzyme activity, and pH) have been extensively investigated [57]. The development of pH-sensitive NPs has received considerable attention, primarily because the slightly acidic environment of the TME can be exploited to develop such NPs [58]. Solid tumors generate substantial amounts of lactate and hydrogen ions from glucose because their rapid glucose catabolism provides sufficient energy for tumor growth [59]. Additionally, lactate and hydrogen ions are generated by nearly all types of solid tumors across various stages of development [60]. Hydrogen ions gradually accumulate in the extracellular and intracellular TME (extracellular pH values of 6.4–6.8 and intracellular pH values of 4.5–5.5), resulting in an acidic TME compared with normal tissues (extracellular pH value of 7.4 and intracellular pH values of 5.0–6.0) [56]. These differences can be exploited to trigger drug release through physical and chemical changes in NPs, allowing their escape from endosomes into the cytoplasm.

### Evaluation of cell uptake, ferroptosis, and cGAS-STING activation in vitro

**Fig. 2:**
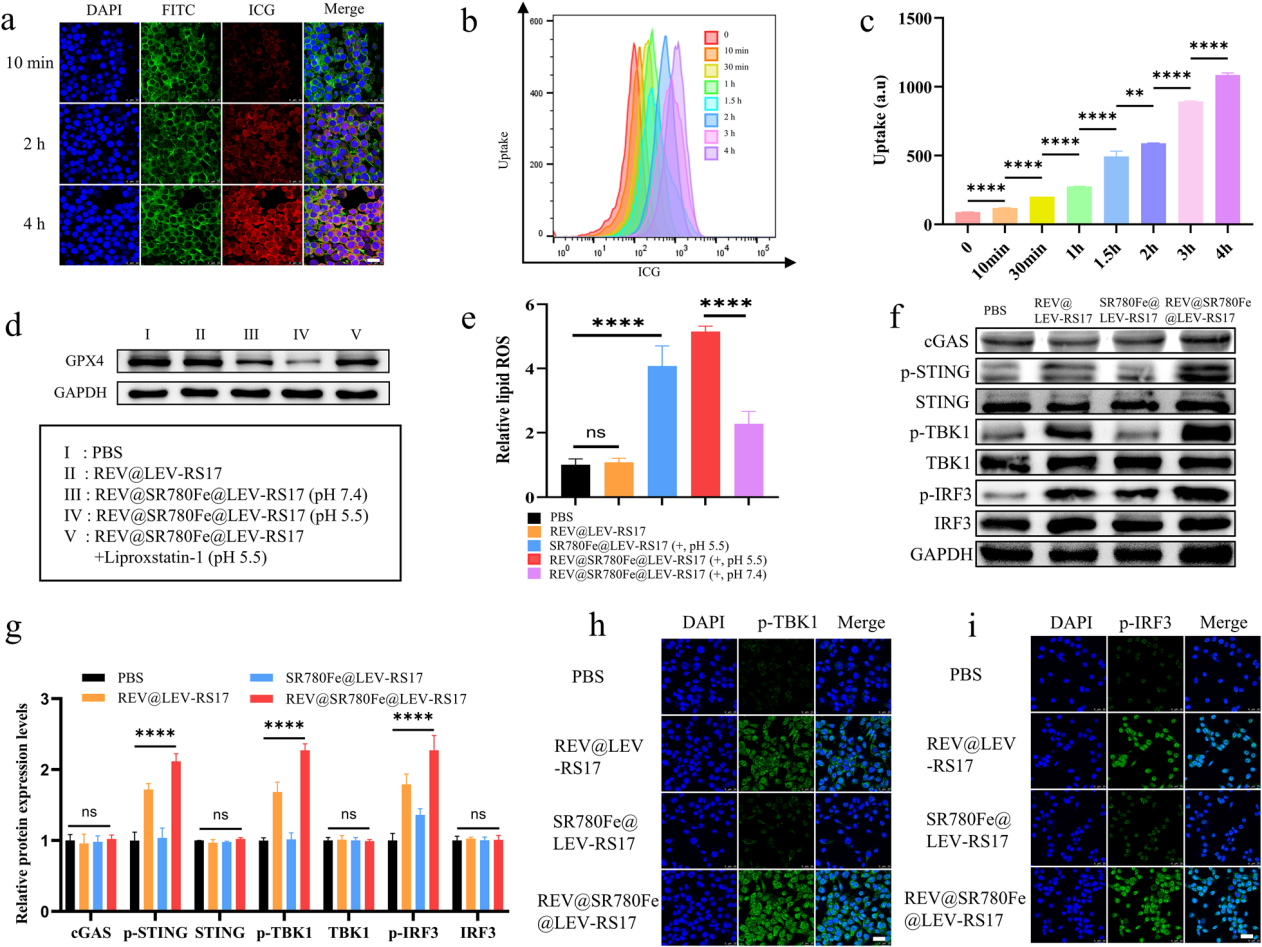
Evaluation of cell uptake, ferroptosis and cGAS-STING activation in vitro. CLSM (**a**), representative FCM (**b**) and quantitative analysis (**c**) of tumor cells after incubation with REV@SR780Fe@LEV-RS17 for different times. Scale bar: 25 μm. **d** WB analysis showing the impact of REV@SR780Fe@LEV-RS17 on the expression of the ferroptosis-related protein. **e** Examination REV@SR780Fe@LEV-RS17-induced intracellular accumulation of lipid peroxide in 4T1 cells in vitro. Expression of cGAS-STING pathway-associated proteins in 4T1 cells after different treatments (**f**) and corresponding quantification (**g**). CLSM images of the p-TBK1 (**h**) and p-IRF3 (**i**) in 4T1 cells in the indicated conditions

REV@SR780Fe@LEV-RS17 NPs incubated with 4T1 cells for various durations were analyzed by CLSM and FCM. After incubation for 10 min, 2 h, and 4 h, CLSM revealed strong ICG fluorescence signals (Fig. 2a). FCM analysis showed a slight and gradual increase in the cellular uptake of NPs (Fig. 2b and c). WB analysis (Fig. 2d) demonstrated that Fe^2+^ strongly inhibited GPX4 expression in 4T1 cells. Fe^2+^ delivery by REV@SR780Fe@LEV-RS17 NPs suppressed the expression of GPX4 in a manner comparable to free Fe^2+^, indicating that Fe^3+^ was efficiently released from NPs and reduced to Fe^2+^ by GSH. This treatment-induced downregulation of GPX4 was abolished by the ferroptosis inhibitor liproxstatin-1. Additionally, REV@SR780Fe@LEV-RS17 NPs significantly induced approximately 5.2-fold higher (p < 0.0001) intracellular lipid ROS than the control group, indicating that tumor cells were exposed to GPX4-dependent lipid peroxidation by REV@SR780Fe@LEV-RS17 NPs (Fig. 2e).

To confirm cGAS-STING pathway activation, the expression levels of cGAS and its downstream markers, including phosphorylated STING (p-STING), phosphorylated TBK1 (p-TBK1), and phosphorylated IRF3 (p-IRF3) were determined by WB (Fig. 2f and g). The results showed that STING was upregulated in the REV@SR780Fe@LEV-RS17 NPs group, confirming that REV could elicit robust STING activation. Furthermore, the levels of p-STING, p-TBK1, and p-IRF3, dependent on an cGAS-STING pathway, were substantially upregulated in the REV@SR780Fe@LEV-RS17 NPs group compared with other groups. Type I IFNs (especially IFN-β) are downstream effectors of the cGAS-STING pathway and generally assumed to function as a bridge between innate and adaptive immunity; they contribute to DCs maturation and migration, as well as the enhancement of CTLs-mediated cytotoxicity [61]. Our data suggest that REV@SR780Fe@LEV-RS17 NPs can target tumor cells and release REV within those cells, activating the cGAS/STING pathway and leading to robust antitumor immune activation. CLSM images of intracellular p-TBK1 and p-IRF3 revealed trends consistent with the WB findings (Fig. 2h and i). REV, an artificially synthesized small molecule purine derivative (chemical formula: C_21_H_27_N_7_O), can act as an inhibitor of the mitotic spindle checkpoint enzyme monopolar spindle 1 (MPS1) and cause DNA damage, thereby activating the cGAS-STING pathway. In a previous report, Christy et al. demonstrated that the cGAS-STING pathway could be activated by REV and AZD1775, then used to investigate chromosomal instability [46]. In the present study, we compared the abilities of different drugs to activate the cGAS-STING pathway; we found that, at the same dose, REV had the greatest activating effect on key proteins downstream of the cGAS-STING (Fig. S3). Therefore, we utilized REV activation of the cGAS-STING pathway for our antitumor analyses.

### PDT-induced ICD effect mediated by REV@SR780Fe@LEV-RS17 NPs

**Fig. 3:**
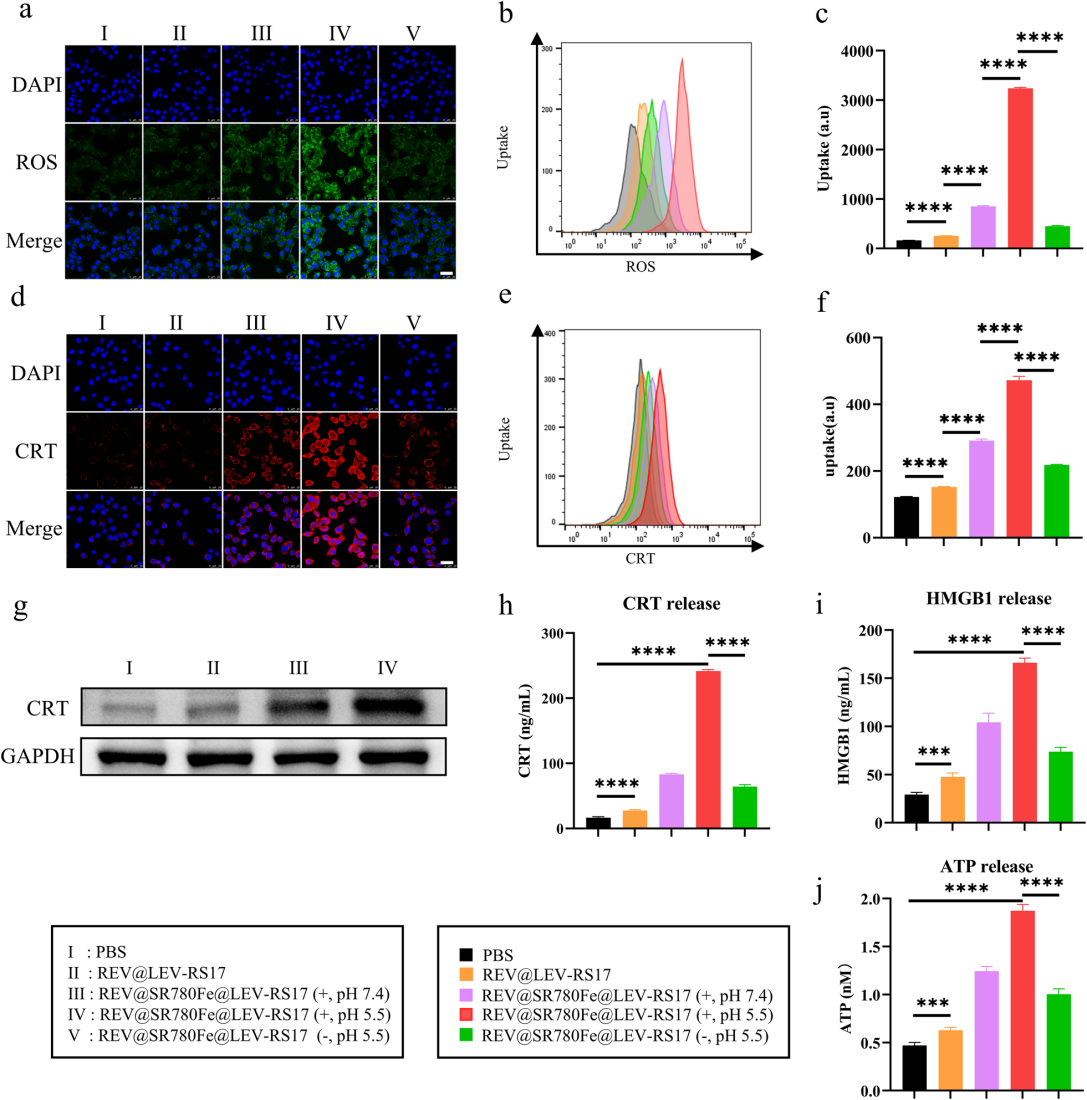
PDT-induced ICD effect based on REV@SR780Fe@LEV-RS17. CLSM images (**a**), representative FCM (**b**) and quantification analysis (**c**) of ROS generation in cells with various treatments. Scale bar: 25 µm. CLSM images (**d**), representative FCM (**e**) and quantification analysis (**f**) of CRT exposure release following various treatments (n = 4). Scale bar: 25 µm. (**g**) WB analysis of CRT proteins expression after different treatments. Release of CRT (**h**), HMGB1 (**i**) and ATP (**j**) in the supernatant from different treatments applied to 4T1 cells (n = 4)

Intracellular ROS levels in 4T1 cells treated with various drugs were examined using DCFH-DA. CLSM showed that green fluorescence was strongest in 4T1 cells treated with REV@SR780Fe@LEV-RS17 NPs (+, pH 5.5), indicating that the NPs induced the highest level of ROS production (Fig. 3a). FCM analyses (Fig. 3b and c) showed that treatment with REV@SR780Fe@LEV-RS17 NPs (+, pH 5.5) increased the ROS levels in 4T1 cells by 3.8-fold compared with REV@SR780Fe@LEV-RS17 NPs (+, pH 7.4) and by approximately 7.1-fold compared with REV@SR780Fe@LEV-RS17 NPs (−, pH 5.5). CLSM also revealed that REV@SR780Fe@LEV-RS17 NPs (+) greatly enhanced the extracellular efflux of CRT protein, further supporting the induction of PDT-mediated ICD in 4T1 cells (Fig. 3d). FCM analysis indicated that the surface CRT levels on tumor cells were slightly increased by SR780Fe (+)-induced PDT. Conversely, simultaneous production of PDT-triggered ROS and GPX4 inhibition-induced lipid peroxidation led to 3.9-fold higher CRT expression in the REV@SR780Fe@LEV-RS17 NPs (+) group than in the control group, indicating that these processes synergistically promoted tumor cell ICD (Fig. 3e and f). Next, we investigated whether REV@SR780Fe@LEV-RS17 NPs could induce ICD and release DAMPs, which are distress signals secreted by tumor cells during ICD. Under stress, the cytoplasmic protein CRT is translocated to the cell surface, where it can recognize and bind to CD91 on DCs, activating them and promoting the downstream maturation of CTLs [62]. WB analysis indicated that REV@SR780Fe@LEV-RS17 NPs (+, pH 5.5) significantly increased CRT release (Fig. 3g) in an 808 nm laser-dependent manner (Fig. S4), indicating that the ICD effect was induced by PDT. As illustrated in Fig. 3h and i, REV@SR780Fe@LEV-RS17 NPs (+, pH 5.5) induced significantly (p < 0.0001) higher levels of CRT and HMGB1 release compared with REV@SR780Fe@LEV-RS17 NPs (+, pH 7.4) and REV@SR780Fe@LEV-RS17 NPs (−, pH 5.5), respectively. Similarly, ATP levels were highest in the REV@SR780Fe@LEV-RS17 NPs (+, pH 5.5) group (Fig. 3j). HMGB1 protein binds to antigen-presenting cells in a cytokine-like manner, resulting in protective immunity in the extracellular space. Furthermore, extracellular ATP release triggers activation of the NLRP3 inflammasome, facilitating the recruitment and activation of antigen-presenting cells for effective CTLs activation. Collectively, these results demonstrate that the PDT strategy using REV@SR780Fe@LEV-RS17 NPs elicits ICD by enhancing the exposure and release of DAMPs.

### In vitro antitumor activity and immune response

**Fig. 4:**
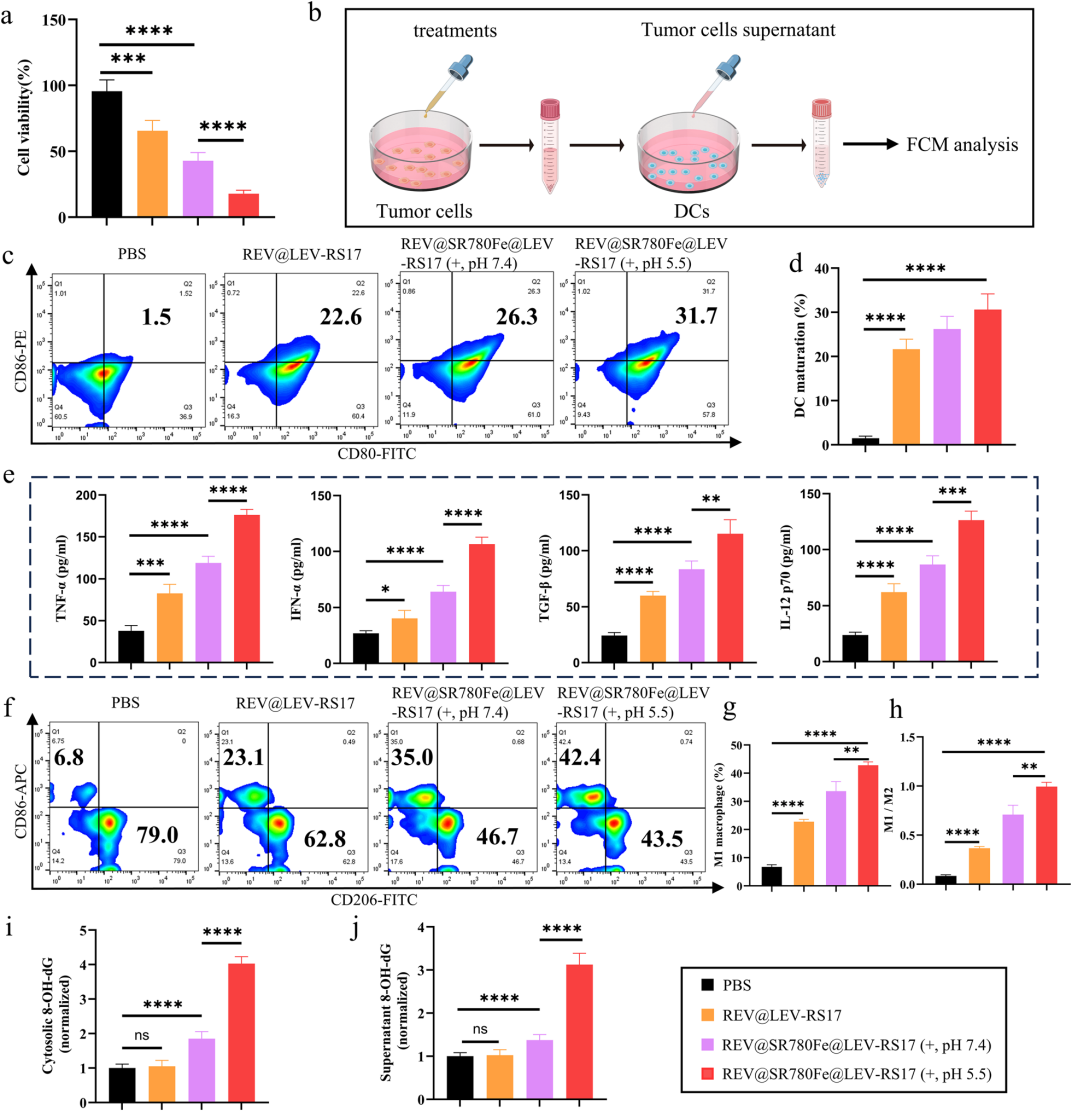
In vitro anti-tumor activity and immune response. **a** The relative viability of cells after incubation with different NPs. **b** Illustration of DCs maturation by different treatments. Representative FCM **c** and quantitative analysis **d** of DCs maturation after different treatments. **e** Cytokine levels of TNF-α, IFN-β, TGF-β and IL-12 p70 in the supernatant from DCs following a variety of treatments tested by ELISA technology (n = 4). Representative FCM (**f**) and quantitative analysis (**g, h**) of repolarization of macrophages in response to various treatments. The amounts of 8-OH-dG in the cytosolic (**i**) and supernatants (**j**) of various treatments applied to 4T1 cells were quantified according to the manufacturer’s protocol (n = 3)

The cytotoxicity of the designed nano-systems was evaluated using CCK-8 assays. HUVECs, L929 cells, L-02 cells, and 4T1 cells, used to investigate the cytotoxicity of blank LEV-RS17, revealed excellent biocompatibility at concentrations of ≤ 40 μg/mL (Fig. S5a). Specifically, the CCK-8 assay demonstrated viabilities of 95.6% ± 8.6%, 65.6% ± 7.9%, 42.8% ± 6.3%, and 17.8% ± 2.7% after treatment with PBS, REV@LEV-RS17 NPs, REV@SR780Fe@LEV-RS17 NPs (+, pH 7.4), and REV@SR780Fe@LEV-RS17 NPs (+, pH 5.5), respectively. REV@SR780Fe@LEV-RS17 NPs (+, pH 5.5) significantly (p < 0.0001) reduced the viability of 4T1 cells compared with the other groups (Fig. 4a).

Next, the cytotoxic effects of REV@SR780Fe@LEV-RS17 NPs (+) were assessed in various breast cancer cell lines, including 4T1, MCF-7, and MDA-MB-231 (Fig. S5b). REV@SR780Fe@LEV-RS17 NPs (+) displayed potential tumor-killing ability in all three cell lines. Further investigation showed that PBS (+) did not display any treatment effect (Fig. S6a). REV@SR780@LEV-RS17 NPs (+)-treated 4T1 cells exhibited a viability of 15.8% ± 2.5%, substantially lower than the viability achieved with double or single therapeutic modalities (Fig. S6b), highlighting the enhanced antitumor efficacy of the designed synergistic triple immunotherapy strategy. Overall, the combined treatment with REV@SR780Fe@LEV-RS17 NPs (+) induced ICD, cGAS-STING activation, and ferroptosis, thereby significantly inhibiting the proliferation of 4T1 breast cancer cells compared with the control group. We subsequently investigated whether combination therapy involving REV@SR780Fe@LEV-RS17 NPs (+) could effectively activate the immune system. An illustration of DCs maturation after the different treatments is shown in Fig. 4b. In the presence of DAMPs released by dying 4T1 cells, REV@SR780Fe@LEV-RS17 NPs (+, pH 5.5) induced 31.7% mature DCs, a percentage 1.2-fold higher than that observed with REV@SR780Fe@LEV-RS17 NPs (+, pH 7.4) (Fig. 4c and d). Additionally, after REV@SR780Fe@LEV-RS17 NPs (+) treatment, significantly (p < 0.0001) higher expression levels of cytokines (including TNF-α, IFN-β, TGF-β, and IL-12 p70) were detected in the culture medium (Fig. 4e), compared with the control group. These results indicated that REV@SR780Fe@LEV-RS17 NP-mediated immunotherapy could strongly activate DCs, thereby increasing antitumor responses. M1-derived NPs can inherit some functions of their parent cells, including the ability to remodel the TME by repolarizing M2 macrophages to M1 macrophages [53]. M2 macrophages were treated with different prepared formulations to determine whether REV@SR780Fe@LEV-RS17 NPs (+) could re-educate TAMs to enhance their antitumor activity (Fig. 4f–h and S7). After each treatment, the expression levels of M1 markers (F4/80 and CD86) were analyzed by FCM. Incubation with REV@SR780Fe@LEV-RS17 NPs (+, pH 5.5) effectively repolarized M2 macrophages to the M1 phenotype, with robust tumor-killing activity. Compared with the control group, a significant (p < 0.0001) increase in M1 macrophages was observed in the REV@SR780Fe@LEV-RS17 NPs (+, pH 5.5) group, indicating the potential for REV@SR780Fe@LEV-RS17 NPs (+, pH 5.5) to reprogram the TME. In addition to tumor cell immunogenicity, the immunosuppressive TME contributes to inhibition of the innate immune system. Although various factors contribute to the low immune responsiveness of the TME, suppressive immune cells within tumors represent a major causative factor. Additionally, TAMs exhibit plasticity and can be polarized into either M1 or M2 phenotypes. Upon encountering ROS, mtDNA activates innate immunity by repolarizing TAMs to the proinflammatory M1 phenotype [63]. To elucidate the mechanism underlying mtDNA escape, we analyzed the 8-OH-dG content in the cytoplasmic matrix and supernatant of cells treated with various prepared formulations. Figure 4i and j shows that the REV@SR780Fe@LEV-RS17 NPs (+, pH 5.5) group produced the highest levels of 8-OH-dG.

### Biodistribution and antitumor efficacy of REV@SR780Fe@LEV-RS17 NPs (+) in vivo

**Fig. 5:**
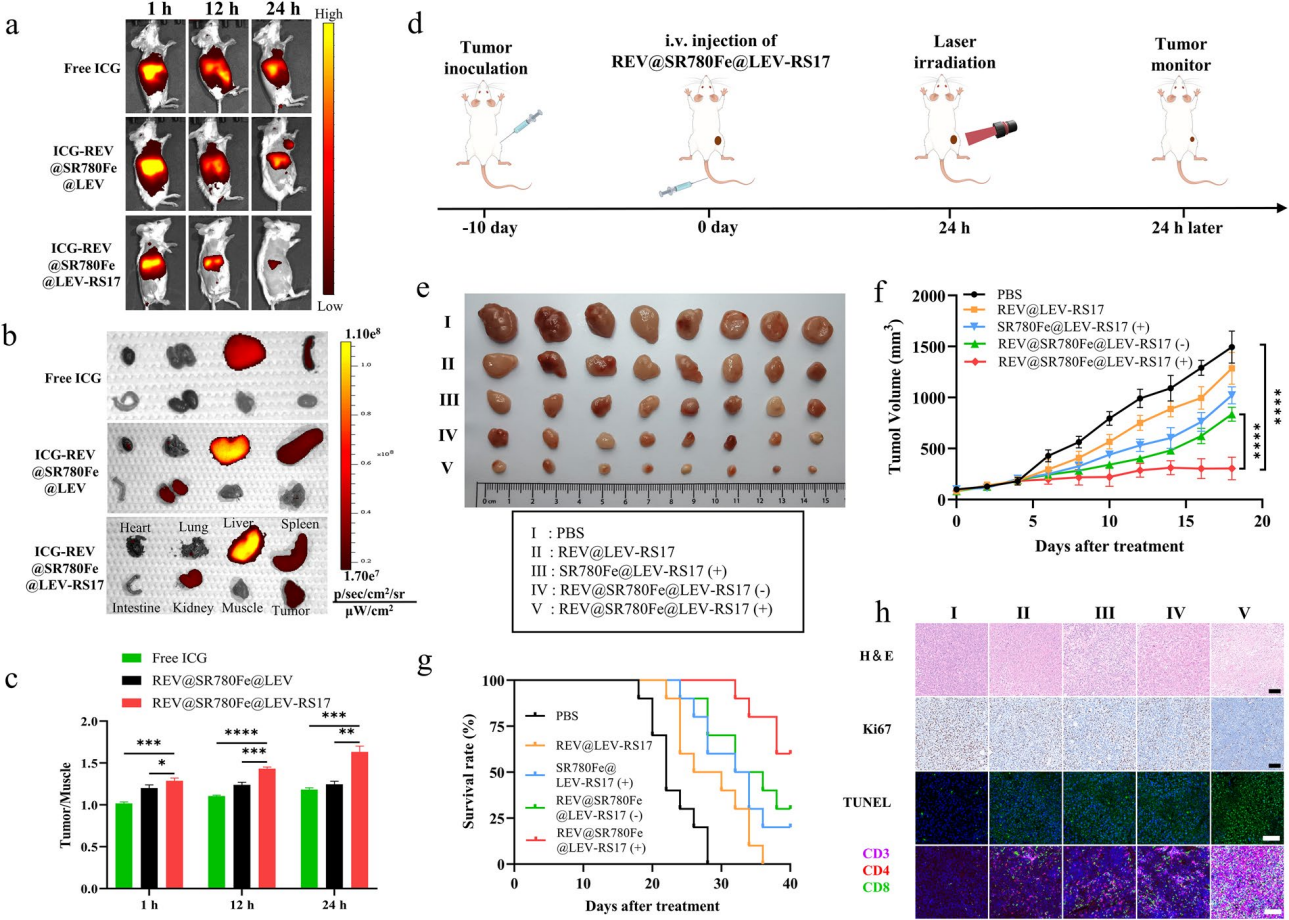
Biodistribution and antitumor efficacy of REV@SR780Fe@LEV-RS17 (+) in vivo. **a** In vivo fluorescence images of 4T1 tumor-bearing mice taken at different time points. **b** Ex vivo fluorescence images of major organs and tumor dissected from mice at 24 h. **c** semi-quantitative analysis of fluorescence images. **d** Treatment schedule of REV@SR780Fe@LEV-RS17 (+). **e** Representative tumor photographs of various treatments for tumor-bearing mice with 4T1 at day 18 (n = 8). The tumor growth (**f**) and survival curves (**g**) of various treatments for tumor-bearing mice with 4T1. **h** H&E, Ki67, TUNEL and CTLs staining of tumors. Scale bar: 100 μm

To investigate the biodistribution of the nanovesicles, 4T1 tumor-bearing mice were intravenously injected with ICG-labeled REV@SR780Fe@LEV or REV@SR780Fe@LEV-RS17 NPs and imaged at various time points (Fig. 5a). Mice injected with ICG-REV@SR780Fe@LEV-RS17 NPs exhibited stronger fluorescence compared with mice that had received ICG-REV@SR780Fe@LEV or free ICG; the maximum fluorescence signal was observed 24 h after injection, indicating the optimal time point for laser-mediated PDT. Ex vivo imaging of major organs and tumor tissues at 24 h post-injection revealed considerably enhanced ICG signal in tumors from the ICG-REV@SR780Fe@LEV-RS17 NPs group (Fig. 5b), indicating that RS17 labeling significantly improved tumor-specific targeting. Furthermore, the tumor/muscle and tumor/liver ratios were higher in the ICG-REV@SR780Fe@LEV-RS17 NPs group than in the ICG-REV@SR780Fe@LEV and free ICG groups (Fig. 5c and S8). Notably, the liver and spleen also exhibited strong fluorescence, likely because of their roles as the main metabolic organs involved in clearing REV@SR780Fe@LEV-RS17 NPs. In solution, negatively charged NPs, such as REV@SR780Fe@LEV-RS17, are generally considered stable without aggregation [64]. Additionally, negatively charged NPs undergo prolonged accumulation in the liver and spleen, possibly due to uptake by macrophages and Kupffer cells.

Next, we subjected REV@SR780Fe@LEV-RS17 NP-mediated immunotherapy to in vivo analysis using the treatment regimen indicated in Fig. 5d. At 24 h after injection of REV@SR780Fe@LEV-RS17 NPs, in vivo antitumor assays were conducted using systemically administered formulations in combination with NIR laser irradiation (808 nm at 1.5 W/cm^2^ for 6 min; SR780Fe and REV both at concentrations of 4 mg/kg). Tumor growth was monitored every other day for 18 days after treatments. Representative tumors were visualized ex vivo upon completion of the efficacy study (Fig. 5e). Compared with the PBS control group, tumor growth was slightly delayed in the SR780Fe@LEV-RS17 NPs (+) and REV@LEV-RS17 (+) groups. Notably, the REV@SR780Fe@LEV-RS17 NPs (+) group exhibited substantial tumor growth suppression (inhibition rate: 79.7%) (Fig. 5f). Mouse survival was monitored for an additional 40 days after treatment (Fig. 5g). As expected, the REV@SR780Fe@LEV-RS17 NPs (+)-treated mice demonstrated the highest survival rate (60%) among all groups. Histopathological examination of the harvested tumors by H&E staining, Ki67 staining, TUNEL staining, and CTLs staining was performed to further evaluate therapeutic efficacy. As shown in Fig. 5h, the most severe changes in cell morphology, the lowest rate of cell proliferation, and the highest rate of cell apoptosis were observed in the REV@SR780Fe@LEV-RS17 NPs (+) group, consistent with the findings regarding tumor volume and survival rate.

### Antitumor immune response activated by REV@SR780Fe@LEV-RS17 NPs (+)

**Fig. 6:**
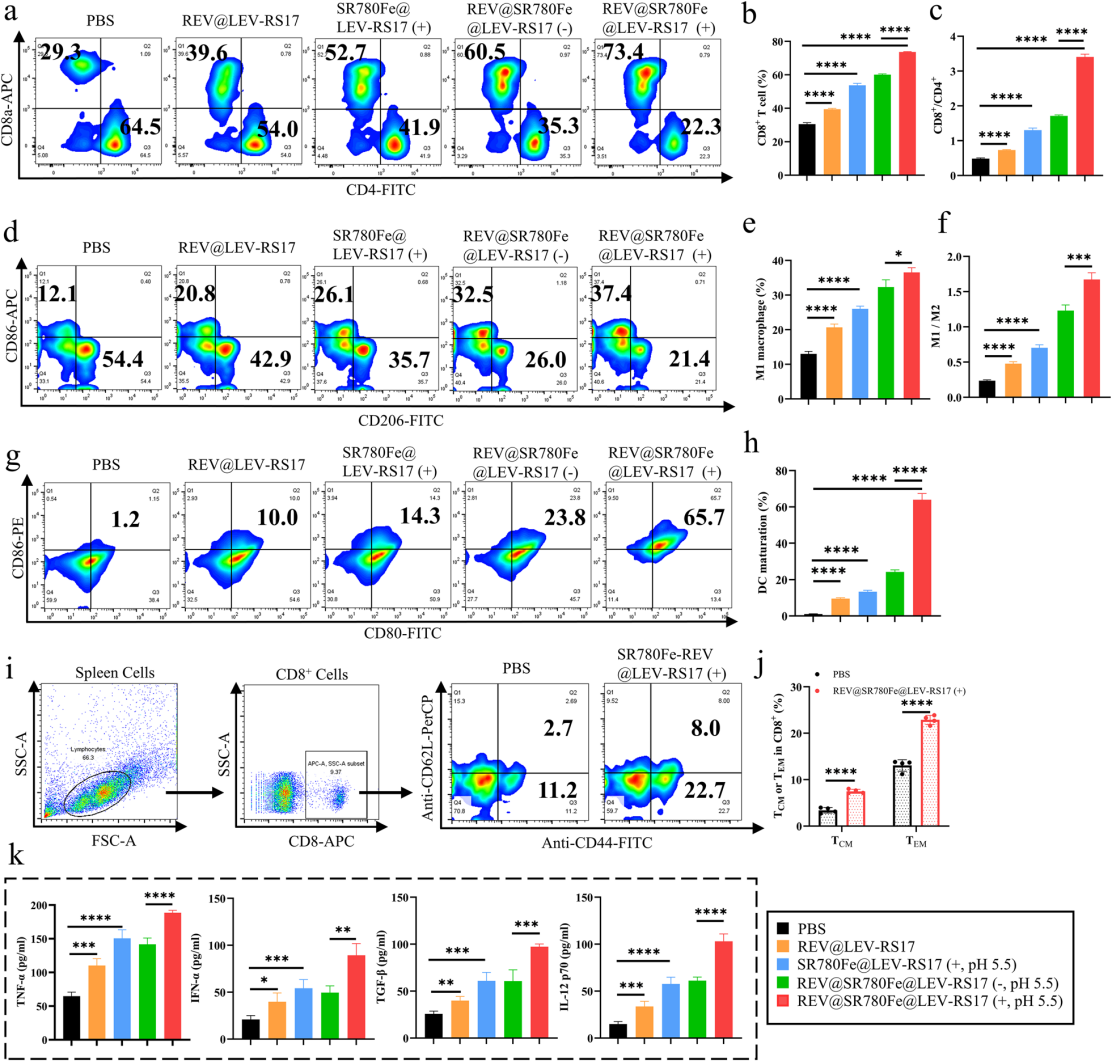
The antitumor immune response activated by REV@SR780Fe@LEV-RS17 (+). Representative FCM (**a**) and quantification analysis (**b, c**) of CTLs in tumor (n = 4). Representative FCM (**d**) and quantification analysis (**e, f**) of macrophage repolarization in tumor. Representative FCM (**g**) and quantification analysis (**h**) of DCs maturation in spleen. Representative FCM (**i**) and quantification analysis (**j**) of CD8^+^CD44^+^CD62L^+^ (T_CM_) and CD8^+^CD44^+^CD62L^−^ (T_EM_) cells proportion in the spleen (n = 4). **k** Based on ELISA technology, Cytokine levels of TNF-α, IFN-β, TGF-β and IL-12 p70 in mice’s serum were determined following different treatments (n = 4)

To evaluate the immune response, spleens, tumors, and serum samples were collected and analyzed. TME infiltration by T lymphocytes, including CD4^+^ and CD8^+^ CTLs capable of killing tumor cells, were also monitored. After treatment with REV@SR780Fe@LEV-RS17 NPs (+), the tumors showed an obvious increase in tumor-infiltrating CTLs (Fig. 6a–c), demonstrating successful induction of antitumor immunity. Considering the excellent TAM polarization results in vitro, we investigated the regulatory effects of REV@SR780Fe@LEV-RS17 NP-mediated treatments on the immunosuppressive TME. As demonstrated in Fig. 6d–f and S9, the proportion of M1 macrophages in tumors significantly increased (p < 0.0001) after treatment with REV@SR780Fe@LEV-RS17 NPs (+), indicating that these NPs could effectively modulate macrophage polarization toward an antitumor M1 phenotype. We performed FCM to examine the maturation of splenic DCs, which play key roles in antigen presentation and T cell activation. Figure 6g and h show that the percentage of mature DCs was significantly greater in the SR780Fe@LEV-RS17 (+) group (13.4% ± 0.8%) than in the PBS group (1.1% ± 0.1%), likely due to the combined effects of ferroptosis and PDT. The REV@SR780Fe@LEV-RS17 NPs (+) group exhibited an even higher percentage of mature DCs (64.0% ± 3.5%) compared with all other groups, consistent with the in vivo antitumor efficacy results. These findings indicated the induction of a strong antitumor immune response, which is expected to prevent tumor metastasis. To assess tumor recurrence prevention, we performed FCM analysis of key splenic immune cells involved in this process: central memory T cells (T_CM_: CD8^+^, CD44^+^, and CD62L^+^) and effector memory T cells (T_EM_: CD8^+^, CD44^+^, and CD62L^−^). We found that T_EM_ and T_CM_ cells were significantly (p < 0.0001) more abundant (1.8-fold and 2.2-fold higher, respectively) in the spleens of mice in the REV@SR780Fe@LEV-RS17 NPs (+) group compared with those in the PBS group (Fig. 6i and j). These results further demonstrated that REV@SR780Fe@LEV-RS17 (+) NPs can effectively kill primary tumor cells and activate the immune system to reduce the risks of tumor metastasis and recurrence. The increased levels of serum cytokines (TNF-α, IFN-β, TGF-β, and IL-12 p70), as measured by ELISAs, indicated that an antitumor immune response had been induced by REV@SR780Fe@LEV-RS17 NPs (+) (Fig. 6k). Among the formulations investigated in this study, REV@SR780Fe@LEV-RS17 NPs (+) demonstrated the strongest effects on DCs maturation, macrophage repolarization, and CTLs infiltration, inducing robust antitumor immunity that resulted in excellent therapeutic and antimetastatic effects.

### Therapeutic efficacy against lung and liver metastases associated with 4T1 in vivo

Metastases, especially in breast cancer, constitute serious problems during antitumor therapy. Metastatic spread and disease progression require a combination of effective targeting strategies. There is increasing evidence that systemic maintenance of tumor suppression involves combination therapies, which are usually more effective than monotherapies in cancer treatment. However, the use of multiple agents and regimens during combination therapy may increase treatment complexity [65]. To address the urgent need for effective yet simple therapies that simultaneously regress primary tumors and prevent metastases, we investigated potent, biocompatible, and multifunctional nanoplatforms with tumor-targeting capacity, pH-activable cytotoxicity, and intrinsic immunity-enhancing effects.

**Fig. 7:**
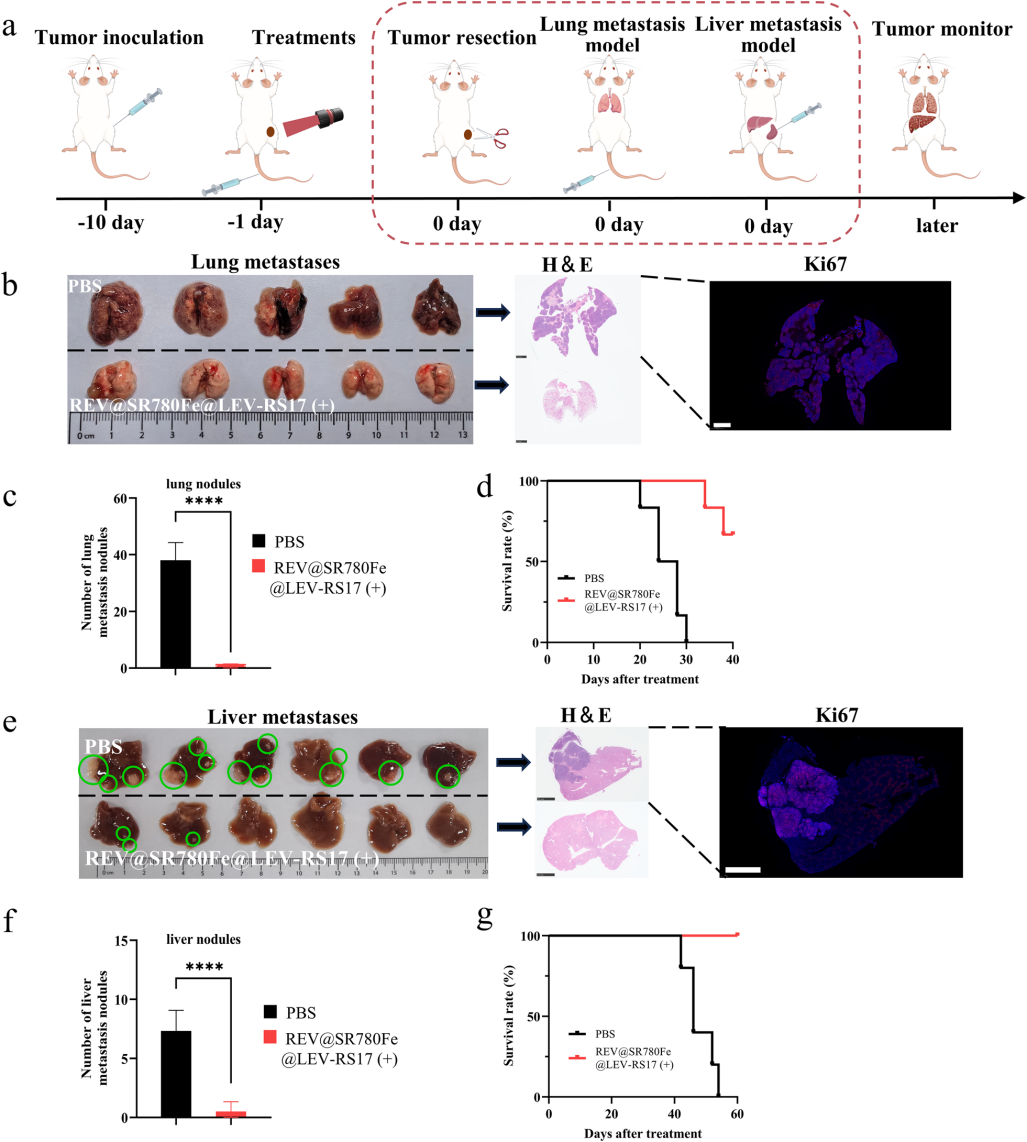
Therapeutic efficacy against lung and liver metastases associated with 4T1 In vivo. **a** Schematic illustration of the treatment plan. For the lung metastasis model, 4T1 cells were injected through the tail vein; for the liver metastasis model, 4T1 cells were injected into the spleen. **b** Images of lung tissues that have been excised and staining of lung sections with H&E and Ki67 on day 40. Scale bar: 2.5 mm. **c** The number of lung nodules associated with metastasis (n = 5). **d** 4T1 lung metastasis mice survival curves after various treatments (n = 5). **e** Images of liver tissues that have been excised and staining of liver sections with H&E and Ki67 on day 60. Scale bar: 2.5 mm. **f** The number of liver metastasis nodules (n = 6). **g** 4T1 liver metastasis mice survival curves after various treatments (n = 6)

Using the 4T1 cell line, we developed lung and liver metastasis models to investigate whether REV@SR780Fe@LEV-RS17 NPs (+) could also effectively inhibit tumor metastasis in mice (Fig. 7a). We found no statistically significant differences in survival rate and or number of nodules between the PBS and PBS (+) groups in both lung metastases (p = 0.43) and liver metastases (p = 0.55); H&E staining confirmed the lack of significant immune activation or therapeutic effects with PBS (+) treatment (Fig. S10). These results showed that irradiation alone did not have any therapeutic benefit, consistent with the in vitro CCK-8 findings (Fig. S6a). In the 4T1 lung metastasis model, BALB/c mice were sacrificed on day 40; their lung tissues were removed and metastatic nodules were quantified. Photographs of the lung metastases are presented in Fig. 7b. Considerably fewer metastatic nodules were detected in the REV@SR780Fe@LEV-RS17 (+) group compared with the PBS group (Fig. 7c). H&E staining and Ki67 staining confirmed the presence and growth of the lung metastases. Additionally, treatment with REV@SR780Fe@LEV-RS17 NPs (+) enhanced the survival rate of tumor-bearing mice from 0% (PBS) to 66.7% (Fig. 7d). In the 4T1 liver metastasis model, BALB/c mice were sacrificed on day 60 and liver tissues were analyzed. Photographs of the collected livers (Fig. 7e) revealed that the control group had metastatic nodules up to approximately 1.1 cm in diameter, whereas the largest nodule in the REV@SR780Fe@LEV-RS17 (+) group reached a diameter of approximately 0.2 cm. The REV@SR780Fe@LEV-RS17 NPs (+) group exhibited significantly fewer metastatic nodules (mean: 1–2) compared with the control group (mean: 7) (Fig. 7f). The survival of tumor-bearing mice was monitored for 60 days. At the end of monitoring, 100% of mice in the REV@SR780Fe@LEV-RS17 NPs (+) group had survived, whereas no mice in the control group had survived (Fig. 7g). These findings demonstrated that REV@SR780Fe@LEV-RS17 NPs (+) offer an effective approach for reducing 4T1 liver metastases through the induction of robust systemic immune responses.

Bioinspired exosome-mimetic NPs have been developed to deliver chemotherapeutic drugs for cancer treatment [66]. For example, Jang et al. [67] generated large quantities of exosome-mimetic NPs from monocytes/macrophages; these NPs exhibited exosome-like characteristics but showed 100-fold higher production yield. They also showed robust antitumor effects and minimal adverse effects, suggesting that bioengineered NPs can serve as novel exosome mimetics for antitumor drug delivery [68]. Similarly, Tang et al. developed innovative nanovesicles by hybridizing M1 macrophage exosomes with phospholipids; these nanovesicles could be used for drug delivery. LEVs containing M1 macrophage exosome membranes are expected to inherit the intercellular communication abilities of M1 macrophages, enhancing drug delivery to tumor cells. Inspired by this strategy, we engineered hybrid nanovesicles containing SR780Fe to form acidity-activatable dynamic hybrid nanoplatforms. Upon modification with RS17 peptide, REV@SR780Fe@LEV-RS17 NPs demonstrated enhanced tumor-targeting ability after systemic administration. Because of their effects on immune system activation, REV@SR780Fe@LEV-RS17 NPs greatly suppressed tumor growth and significantly prolonged survival among BALB/c mice with subcutaneous xenograft tumors, lung metastases, and liver metastases.

**Fig. 8:**
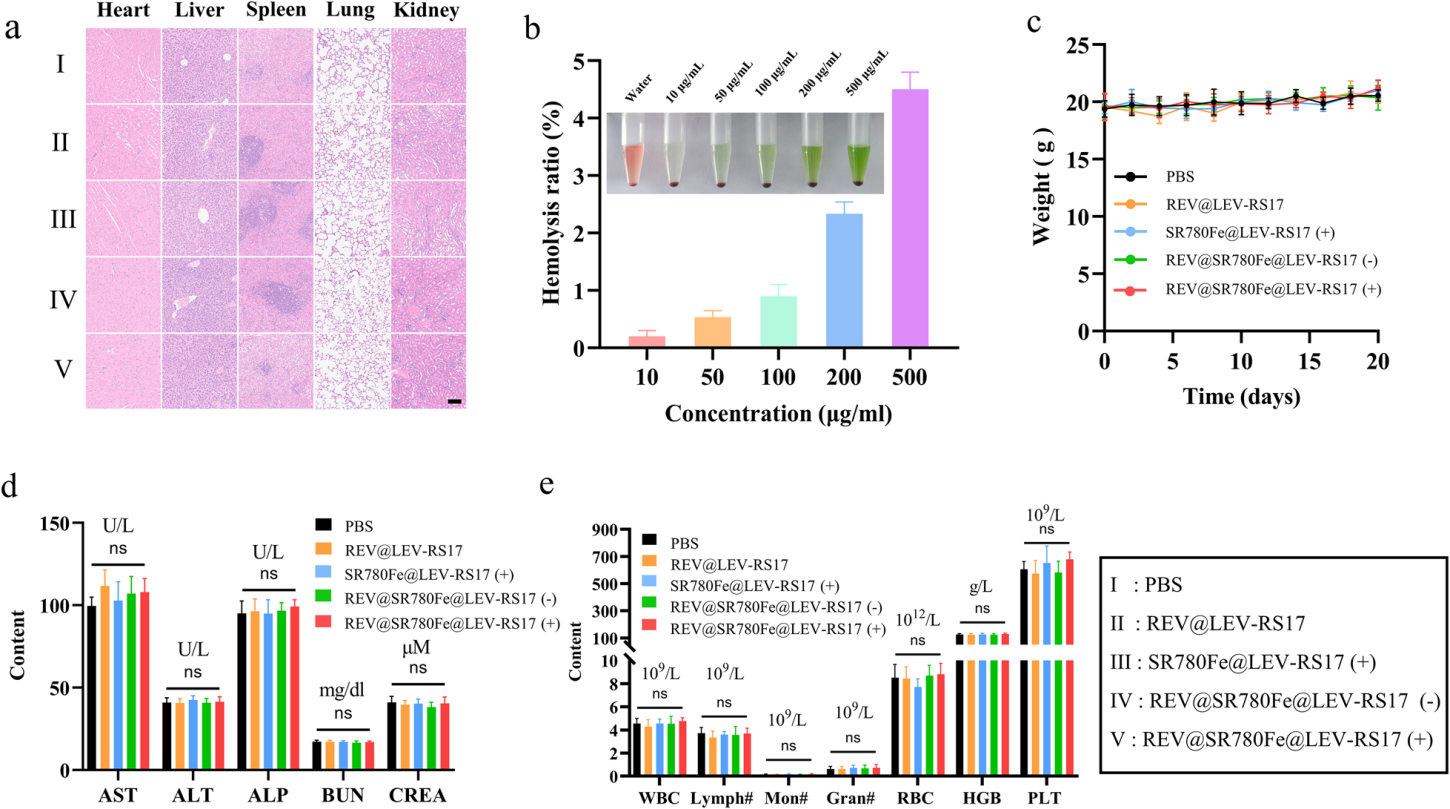
In vivo biosafety test. **a** H&E-stained slice images of major organs. Scale bar: 100 μm. **b** Representative pictures of various concentrations of REV@SR780Fe@LEV-RS17 NPs incubated with 2% erythrocyte suspensions. Ratio of hemolysis at different REV@SR780Fe@LEV-RS17 concentrations (n = 3). Distilled water served as the positive control. **c** Weight changes within 20 days in animals. **d** Measurements of liver function markers and kidney function markers in the blood biochemistry. **e** Major blood routine parameters data

Importantly, no histopathological changes were observed in major organs (Fig. 8a). Hemolysis tests performed to investigate the biosafety of REV@SR780Fe@LEV-RS17 NPs (Fig. 8b) revealed minimal hemolysis, even at a concentration of 500 μg/mL; this value was considerably below the 5% safety threshold [53]. No obvious weight loss was observed in the mice after various treatments (Fig. 8c); serum biochemistry and routine blood test parameters remained normal (Fig. 8d and e), confirming the robust biosafety and biocompatibility of REV@SR780Fe@LEV-RS17 NPs in combination immunotherapy.

This study had some limitations. First, because light has limited tissue penetration capacity, our PDT strategy is not suitable for internal tumors. Second, the long-term toxicity and metabolism of the designed NPs have not been elucidated and warrant further investigation. Finally, this strategy requires extensive dose optimization before it can be tested in larger animal models.

## Conclusion

In this study, we synthesized and designed SR780Fe, a pH-responsive photosensitizer with excellent stability and therapeutic efficacy in PDT. We then developed bioinspired hybrid nanovesicles by fusing M1 EVs with liposomes to co-encapsulate REV and SR780Fe. These nanovesicles were modified with RS17 peptide to increase the tumor-targeting ability of the hybrid nanovesicles. Finally, we used thin film dispersion to prepare pH-responsive active targeting NPs with an “off/on” functionality. SR780-induced ICD, Fe^2+^-induced ferroptosis, and DCs maturation were synergistically achieved by the REV-activated cGAS-STING pathway to stimulate T cell participation in antitumor immunity. Therefore, sustained immunotherapeutic effects were obtained by reprogramming “cold” TMEs. Our observations indicated that the designed REV@SR780Fe@LEV-RS17 (+) NPs inhibited primary tumor growth. Additionally, lung and liver metastases were suppressed, reducing the likelihood of tumor recurrence; this reduced likelihood was demonstrated by the increased percentages of M1-like TAMs and CTLs within tumors, as well as mature DCs in the spleen. Additionally, the establishment of a persistent immune memory response protected the mice from subsequent tumor challenge. The synergistic combination of PDT, ferroptosis, and cGAS-STING pathway activation led to enhanced tumor treatment effects. In the future, this strategy may allow synergistic immunotherapies to be developed much more rapidly than the current rate.

### Supplementary Information

Additional file 1.

## Data Availability

The analyzed datasets generated during this study are available from the corresponding author on reasonable request.

## Abbreviations

TME: Tumor microenvironment
EVs: Extracellular vesicles
LEVs: Hybrid nanovesicles (liposome and EVs)
ICBs: Checkpoint blockades
ICD: Immunogenic cell death
DAMPs: Damage-associated molecular patterns
ATP: Adenosine triphosphate
CRT: Calreticulin
HMGB1: High mobility group box 1
DCs: Dendritic cells
PDT: Photodynamic therapy
ROS: Reactive oxygen species
TAMs: Tumor-associated macrophages
cGAS: Cyclic GMP-AMP synthase
STING: Stimulator of interferon genes
cGAMP: 2′-3′-Cyclic-GMP-AMP
TBK1: TANK-binding kinase 1
IRF3: Interferon regulatory factor 3
IFN: Interferons
GSH: Glutathione
GPX4: Glutathione peroxidase 4
NPs: Nanoparticles
NIR: Near-infrared
CTLs: Cytotoxic T lymphocytes
ICG: Indocyanine green
TEM: Transmission electron microscope
UV–vis: Ultraviolet–visible spectrometer
DLS: Dynamic light scattering
CLSM: Confocal laser scanning microscopy
FCM: Flow cytometry
mtDNA: Mitochondrial DNA
ELISA: Enzyme-linked immunosorbent assay
WB: Western blot
H&E: Hematoxylin and eosin
AST: Aspartate aminotransferase
ALT: Alanine aminotransferase
ALP: Alkaline phosphatase
CREA: Creatinine
BUN: Blood urea nitrogen
PDI: Polydispersity index
FBS: Fetal bovine serum
APCs: Antigen-presenting cells
T_CM_: Central memory T cells
T_EM_: Effector memory T cells
