# Supplementary material for: Acidity-activatable dynamic hybrid nanoplatforms derived from extracellular vesicles of M1 macrophages enhance cancer immunotherapy through synergistic triple immunotherapy
Source: J Nanobiotechnology. 2024 Jul 20;22:430. doi: 10.1186/s12951-024-02719-7 (PMC11264854; doi:10.1186/s12951-024-02719-7)
Supplement: Supplementary file 1 — Additional file 1. [file 12951_2024_2719_MOESM1_ESM.docx]

**Supporting information**

**Materials**

DMEM medium, RPMI-1640 medium, phosphate buffer (PBS), fetal bovine serum (FBS), trypsin-EDTA, dimethyl-sulfoxide (DMSO), 100 U/mL penicillin, 100 mg/mL streptomycin, CCK8 kit, BCA kit, mouse TNF-α ELISA kit, mouse IFN-α ELISA kit, mouse TGF-β ELISA kit and mouse IL-12 p70 ELISA kit were obtained from SolarBio (Beijing, China). Reversine was purchased from Macklin Inc. (Shanghai, China). Tris-Glycine SDS Buffer (pH 8.3, 10×), Tris-Glycine Transfer Buffer (pH 8.3, 10×), TBST (pH 8.0, 10×) were obtained from CW Biotech (Beijing, China). RIPA lysis buffer and pheylmethylsulfonyl fluoride (PMSF) were purchased from Beyotime Biotechnology (Shanghai, China).

**Synthesis of SR780Fe**

The synthesis method of SR780Fe refers to previous literature reports^1^. Synthesis of compound A： 2-thiophenethiol (0.263 g, 2.26 mmoL) and 1-methylpiperazine (0.341 g, 3.40 mmoL) were mixed into toluene (8 mL) and stirred at 110℃ for reflux reaction for 8 h. By rotary evaporating the solution, the crude product was obtained, which was then purified by column chromatography (silica gel, dichloromethane). The final product compound A was orange oil.

Synthesis of SR780: Compound A (0.2 g, 1.1 mmoL) and croconic acid (0.062 g, 0.44 mmoL) were dissolved in toluene and n-butanol (30 mL, 1:1) and stirred at 110 ℃ for reflux reaction for overnight. The mixture was filtered and dried, redissolved in methanol and then purified by column chromatography (C18, methanol and deionized water). SR780 was obtained as black powder after the solvent was removed under vacuum.

Synthesis of SR780Fe: Add FeCL3·6H2O (1.48 mg) and SR780 (3.17 mg) to 5 mL of methanol and stir for 2 minutes at room temperature. In the above solution, 100 mg of DSPE-PEG_2000_ was fully dissolved. After the solvent was removed by thin-film dispersion method, ultrapure water (3 mL) was slowly added and ultrasonic dispersion was performed to obtain SR780Fe.

**Preparation of REV@SR780Fe@Lip nanoparticles**

REV@SR780Fe@Lip NPs were developed by the conventional thin film-hydration method. Briefly, lipids with a ratio of DSPC/DSPE-PEG2000/Chol of 90:10:4 (mol/mol/mol) were dissolved in chloroform at 20℃ for 30 min. Afterwards, REV and SR780Fe solutions (diluted in 4 mL PBS) were used to hydrate the film at 20℃ for 20 min. Finally, REV@SR780Fe@Lip was harvested after probe ultrasonication and dialysis.

**Isolation of M1 EVs**

M1 EVs were isolated according to the published protocols^2^. Detailedly, a 48-hour treatment with lipopolysaccharide (LPS) polarized RAW 264.7 cells to the M1 phenotype. Afterwards, the phenotype of macrophages was evaluated via flow cytometry (FCM). To collect M1 EVs, 1×10^7^ polarized RAW 264.7 cells were cultured for 48 h in 20 mL DMEM medium without exosomes. Following that, the cell culture medium was harvested by centrifugation. Cells and their debris were removed by centrifugating at 12000 ×g for 30 min at 4℃. Then, the supernatant was ultracentrifugated at 120000 ×g for 120 min at 4℃ (Beckman, USA). Next, the supernatant was carefully pipetted out and the obtained pellet in the bottom was washed by ice-cold PBS and ultracentrifugated again at 120000 ×g for 120 min at 4℃. Finally, the precipitate containing M1 EVs was resuspended in ice-cold PBS and the protein concentration of them was calculated by BCA method according to the manufacture’s protocol.

1 Sun, R. *et al.* pH-activated nanoplatform for visualized photodynamic and ferroptosis synergistic therapy of tumors. *Journal of Controlled Release : Official Journal of the Controlled Release Society* **350**, 525-537, doi:10.1016/j.jconrel.2022.08.050 (2022).

2 Tang, L. *et al.* Extracellular Vesicles-Derived Hybrid Nanoplatforms for Amplified CD47 Blockade-Based Cancer Immunotherapy. *Advanced Materials (Deerfield Beach, Fla.)*, e2303835, doi:10.1002/adma.202303835 (2023).

Table S1. Antibodies used in the study.

| Antibodies | Source | Catalog |
| --- | --- | --- |
| iNOS | Abclonal | A3774 |
| TSG101 | Abclonal | A2216 |
| CD63 | Abclonal | A5271 |
| CD9 | Abclonal | A19027 |
| GPX4 | Abclonal | A1933 |
| Anti-mouse GAPDH | Abclonal | AC001 |
| HRP Goat Anti-Rabbit IgG(H+L) | Abclonal | AS014 |
| Anti-mouse cGAS | Abclonal | A8335 |
| Anti-mouse p-STING | Abclonal | AP1369 |
| Anti-mouse STING | Abclonal | A3575 |
| Anti-mouse p-TBK1 | Abclonal | AP0847 |
| Anti-mouse TBK1 | Abclonal | A3458 |
| Anti-mouse p-IRF3 | Abclonal | AP0857 |
| Anti-mouse IRF3 | Abclonal | A2172 |
| Anti-mouse CRT | Abclonal | A1066 |
| Abflo 594-conjugated Goat Anti-Rabbit IgG(H+L) | Abclonal | AS039 |
| FITC Goat Anti-Mouse IgG (H+L) | Abclonal | AS001 |
| APC Anti-Mouse CD86 | Elabscience | E-AB-F0994UE |
| FITC Anti-Mouse CD206 | Elabscience | E-AB-F1135C |
| PE Anti-Mouse CD86 | Elabscience | E-AB-F0994UD |
| FITC Anti-Mouse CD80 | Elabscience | E-AB-F0992UC |
| PE Anti-Mouse F4/80 | Elabscience | E-AB-F0995D |
| PerCP Anti-Mouse CD62L | Elabscience | E-AB-F1011J |
| FITC Anti-Mouse CD44 | Elabscience | E-AB-F1100C |
| PE anti-mouse CD3 | BioLegend | 100205 |
| FITC anti-mouse CD4 | BioLegend | 100405 |
| APC anti-mouse CD8a | BioLegend | 100711 |


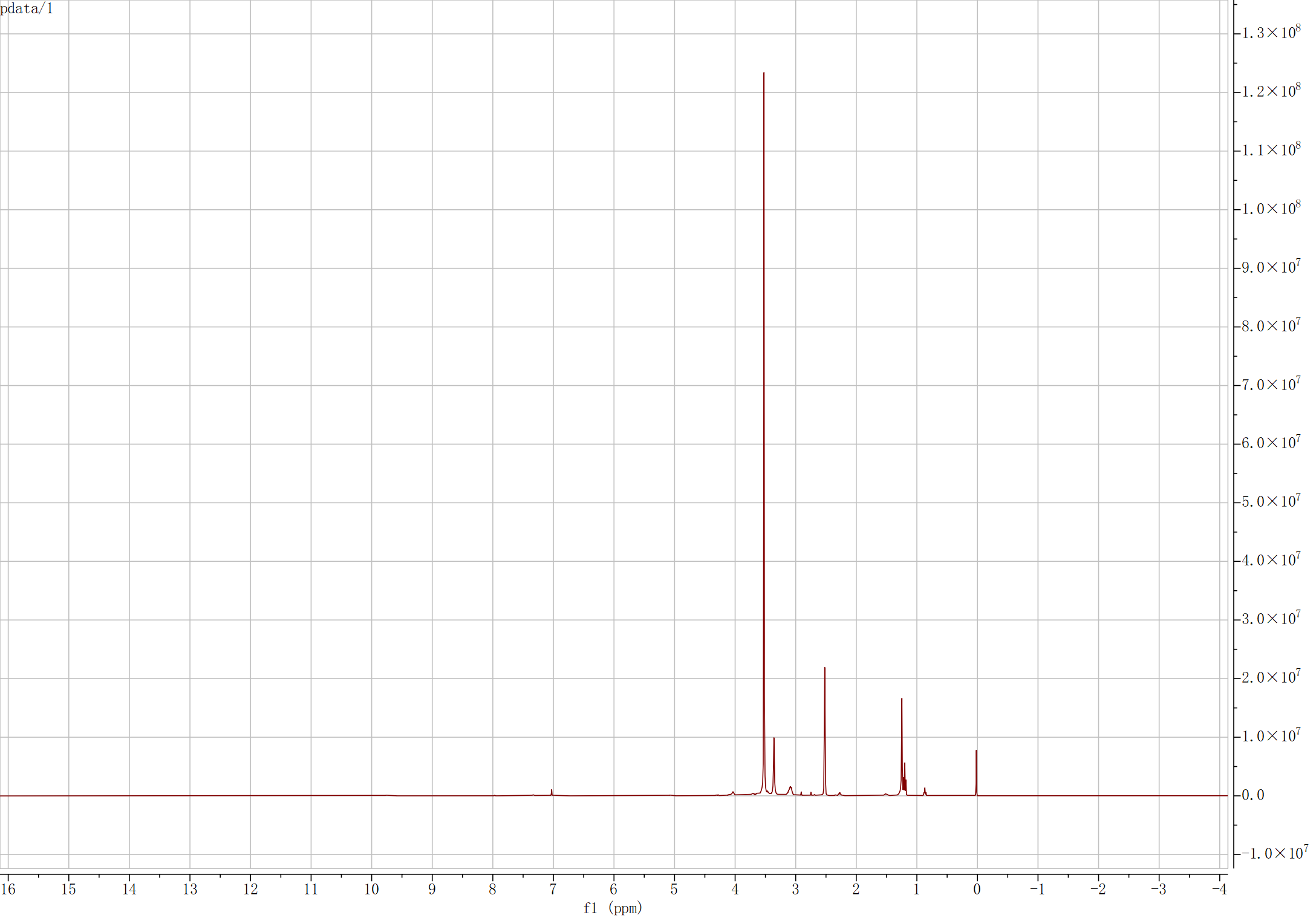


Fig. S1 13C NMR spectrum of SR780Fe.





Fig. S2 Characterization of REV@LEV-RS17 and REV@SR780Fe@LEV. TEM images (a) and size distribution(b) of REV@LEV-RS17 and REV@SR780Fe@LEV. (c) WB analysis of the protein biomarkers expression in REV@LEV-RS17 and REV@SR780Fe@LEV NPs. The variation of DLS (d), Zeta potential (e) and PDI (f) of REV@LEV-RS17 and REV@SR780Fe@LEV NPs dispersed in FBS in 7 days.





Fig. S3 Expression of cGAS-STING pathway-associated proteins in 4T1 cells after different treatments (a) and corresponding quantification (b).


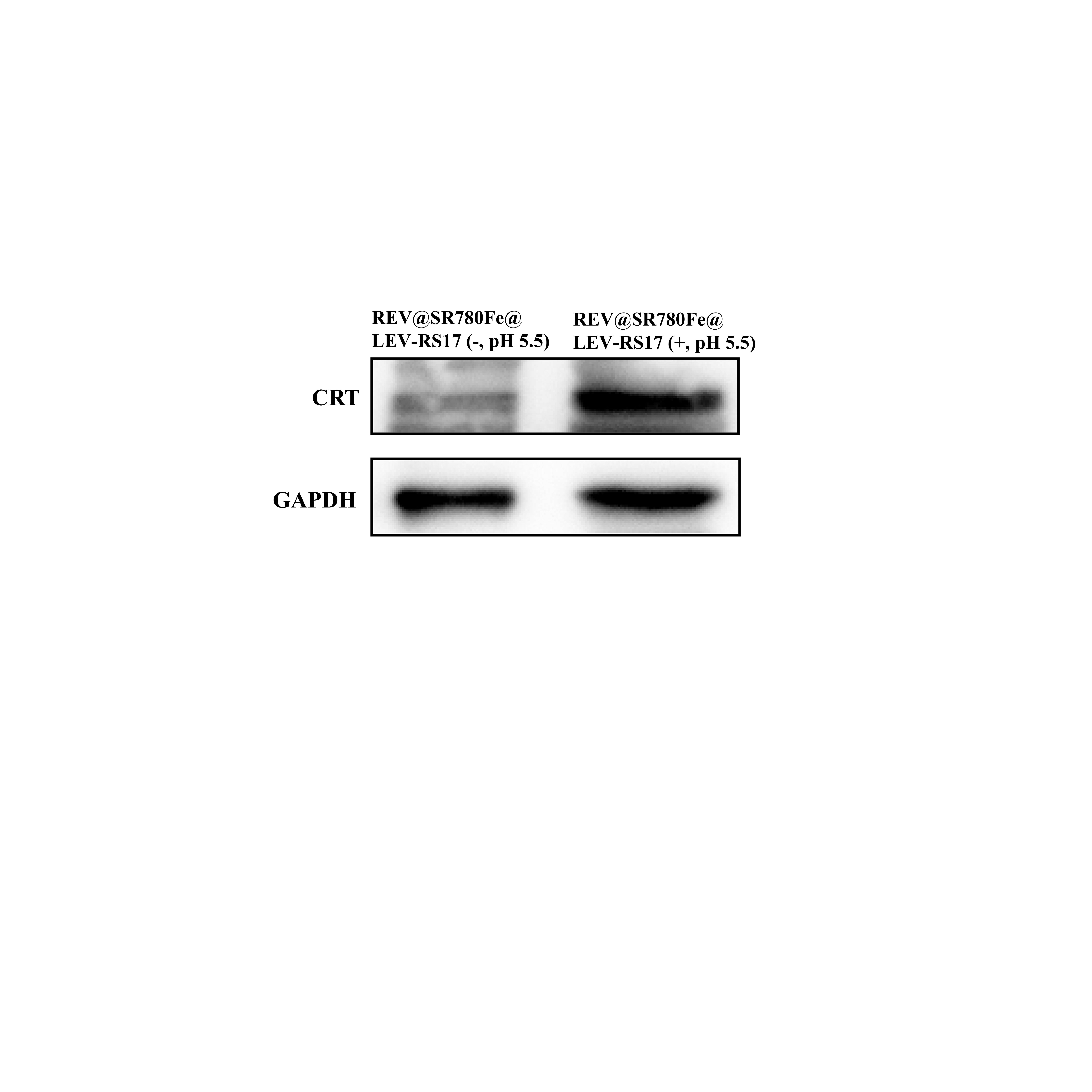


Fig. S4 WB analysis of CRT proteins expression after different treatments.


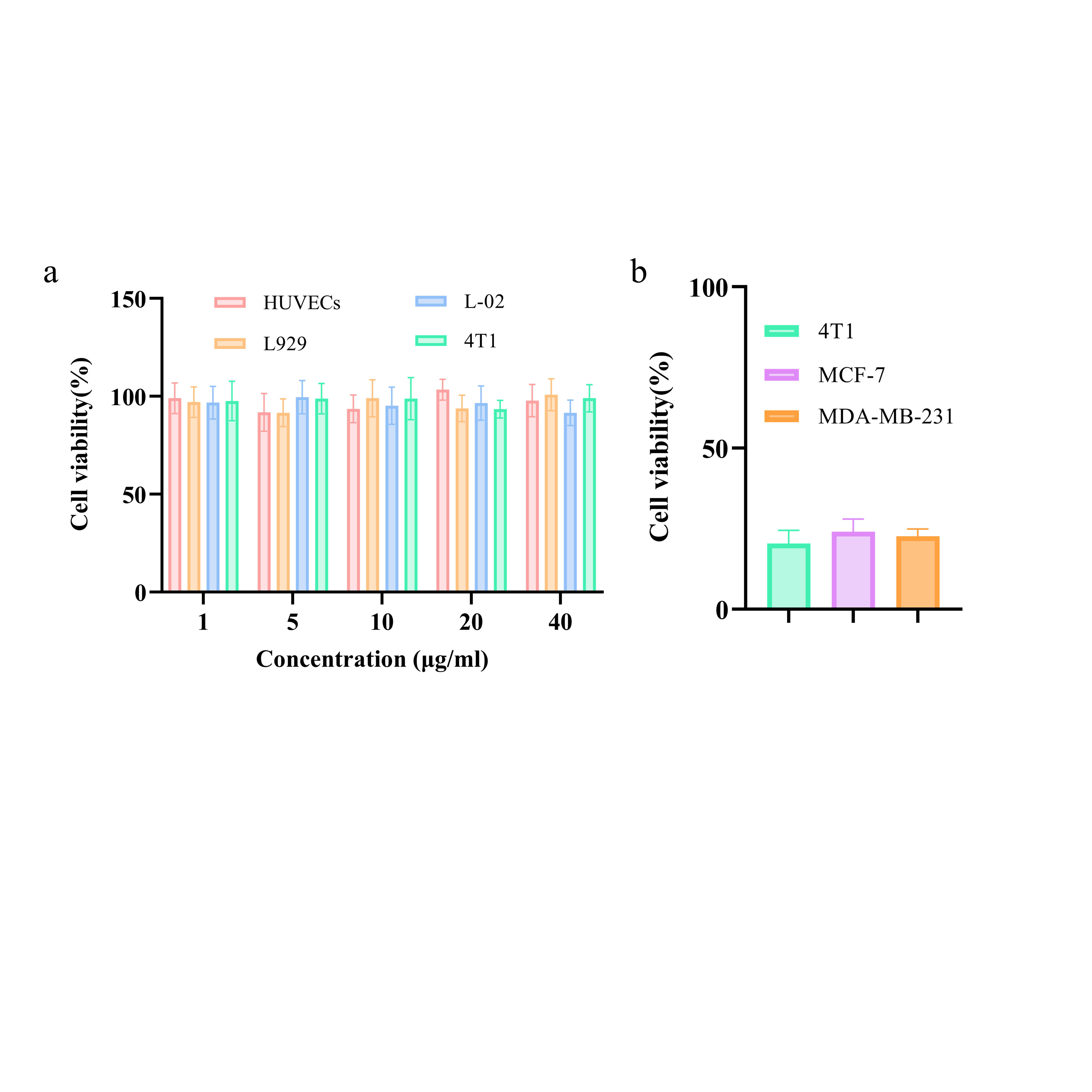


Fig. S5 (a) Relative cell viability after incubation with blank LEV-RS17 NPs with different concentrations. (b) Relative cell viability after incubation with different breast cancer cell lines.





Fig. S6 (a) The relative viability of cells with or without irradiation. (b) The relative viability of cells after incubation with different NPs.


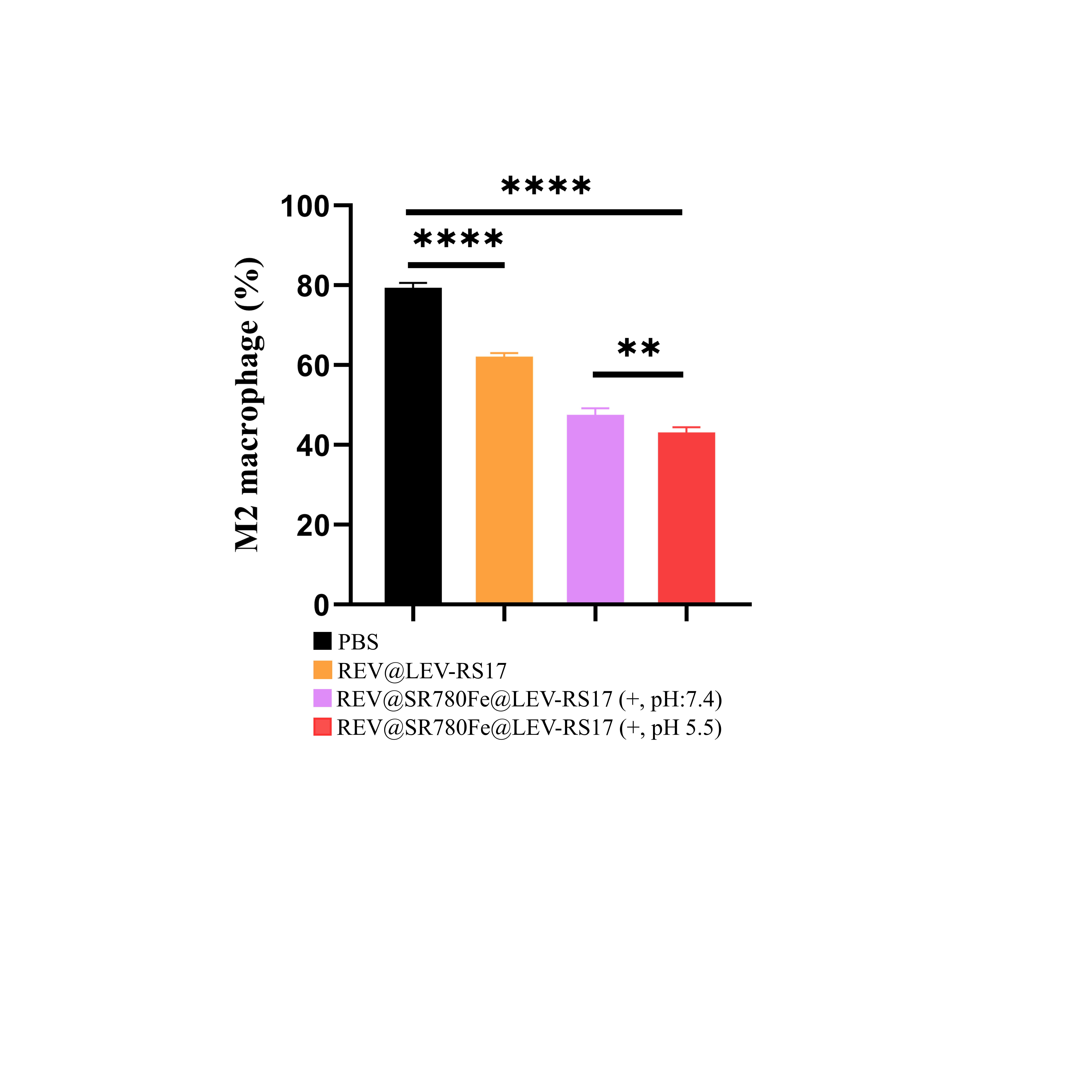


Fig. S7 In vitro quantification analysis of macrophage repolarization in tumor.


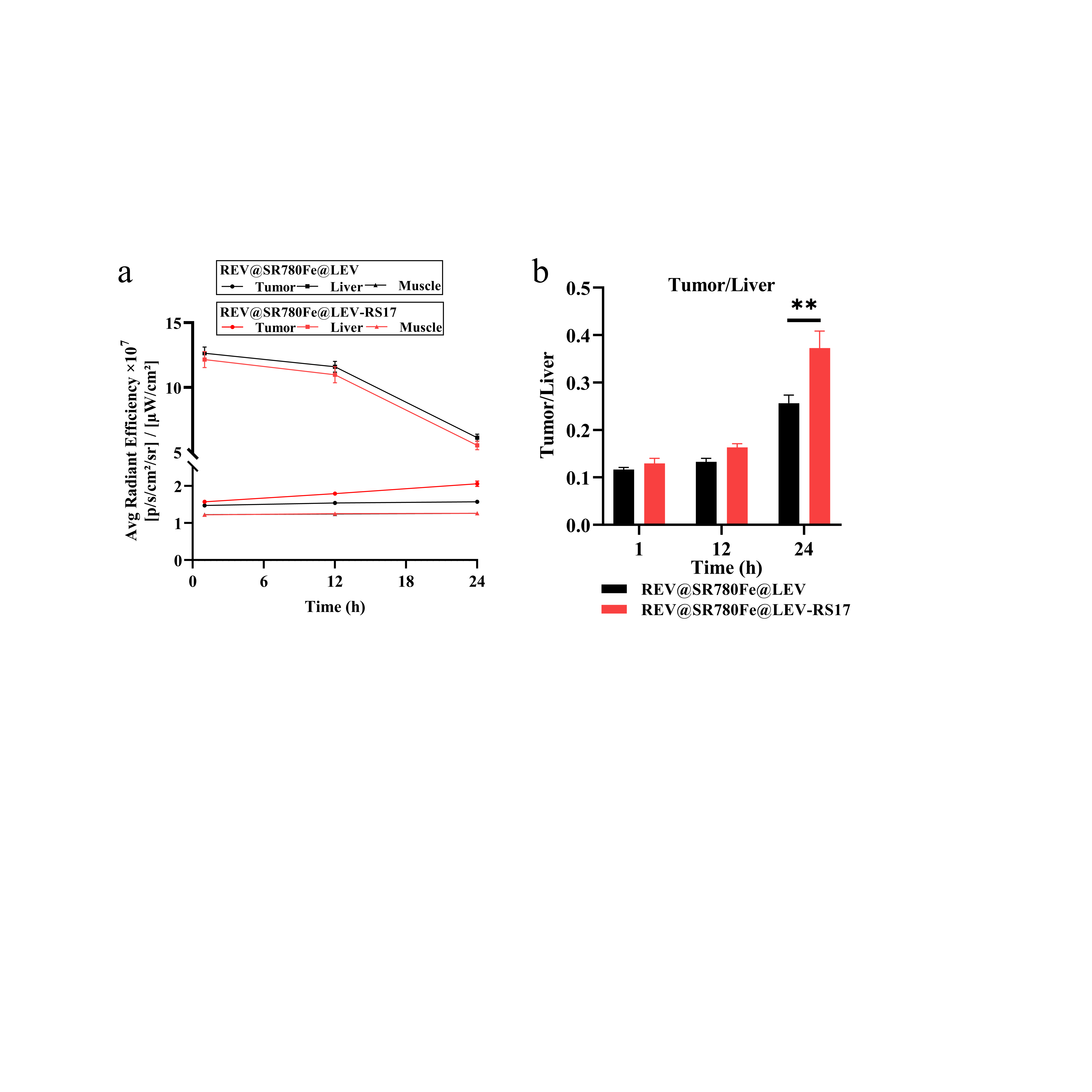


Fig. S8 (a) Semi-quantitative analysis of fluorescence images at different time points. (b) tumor/liver ratio at different time points.


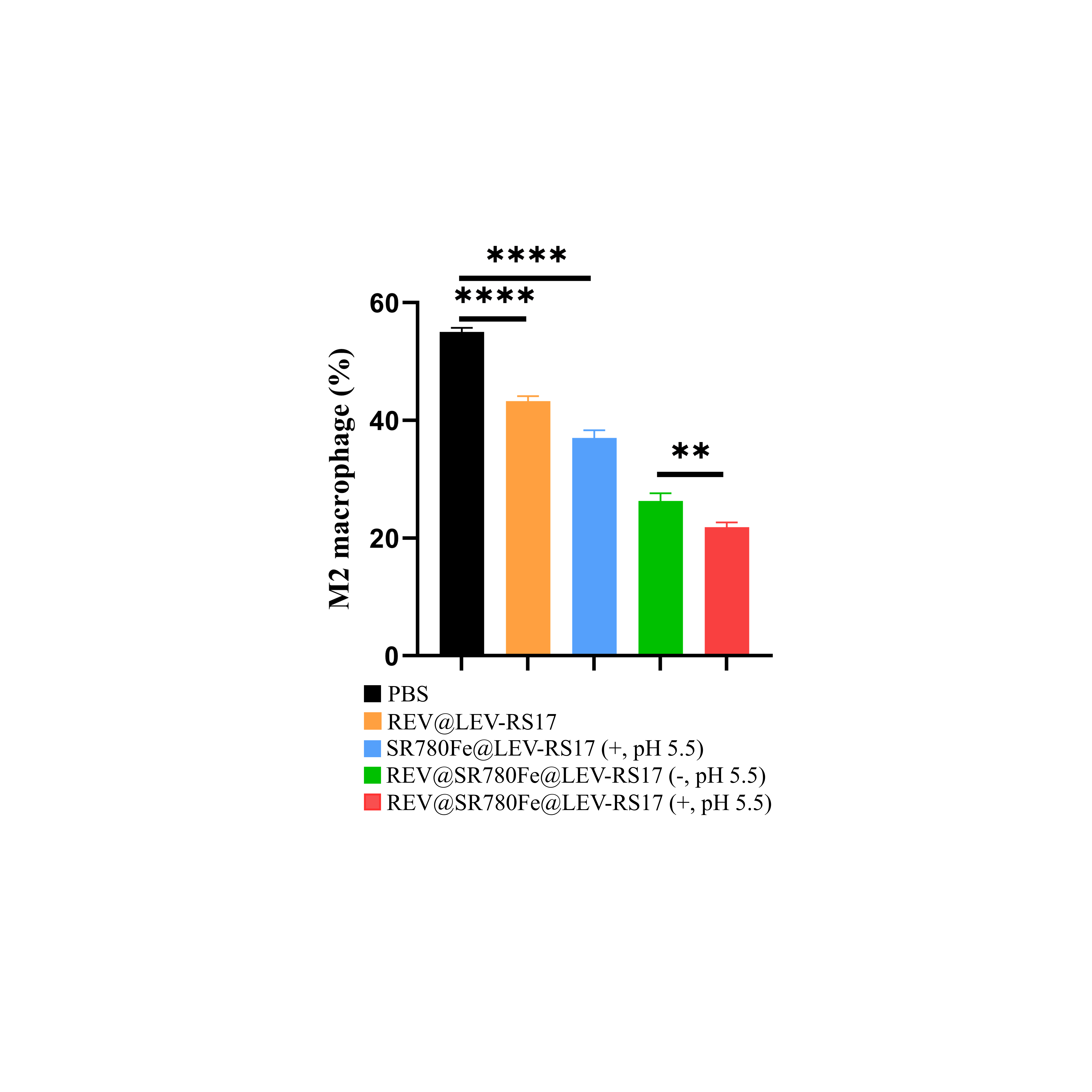


Fig. S9 In vivo quantification analysis of macrophage repolarization in tumor.

Fig. S10 (a) Images of lung tissues that have been excised and staining of lung sections with H&E (b) on day 28. Scale bar: 2.5 mm (left), 100 μm (right). (c) The number of lung nodules associated with metastasis (n = 3). (d) 4T1 lung metastasis mice survival curves after various treatments (n = 3). (e) Images of liver tissues that have been excised and staining of liver sections with H&E and Ki67 on day 50. Scale bar: 2.5 mm (left), 100 μm (right). (f) The number of liver metastasis nodules (n = 3). (g) 4T1 liver metastasis mice survival curves after various treatments (n = 3).
